# Up-regulation of gap junction in peripheral blood T lymphocytes contributes to the inflammatory response in essential hypertension

**DOI:** 10.1371/journal.pone.0184773

**Published:** 2017-09-14

**Authors:** Xin Ni, Ai Wang, Liang Zhang, Li-ya Shan, Hai-chao Zhang, Li Li, Jun-qiang Si, Jian Luo, Xin-zhi Li, Ke-tao Ma

**Affiliations:** 1 Department of Physiology, Medical College of Shihezi University, Shihezi, Xinjiang, China; 2 Key Laboratory of Xingjiang Endemic and Ethnic Diseases, Medical College of Shihezi University, Shihezi, Xinjiang, China; 3 The First Affiliated Hospital of Xinjiang Medical University, Urumqi, Xinjiang, China; 4 Department of Pathophysiology, Medical College of Shihezi University, Shihezi, Xinjiang, China; Rutgers University, UNITED STATES

## Abstract

Inflammation has been shown to play an important role in the mechanisms involved in the pathogenesis of hypertension. Connexins (Cxs)-based gap junction channels (GJCs) or hemichannels (HCs) are involved in the maintenance of homeostasis in the immune system. However, the role of Cx43-based channels in T-lymphocytes in mediating the immune response in essential hypertension is not fully understand. The present study was designed to investigate the role of Cxs-based channels in T lymphocytes in the regulation of hypertension-mediated inflammation. The surface expressions of T lymphocyte subtypes, Cx40/Cx43, and inflammatory cytokines (IFN-γ (interferon-gamma) and TNF-ɑ (tumor necrosis factor alpha)) in T cells, as well as gap junction communication of peripheral blood lymphocytes from essential hypertensive patients (EHs) and normotensive healthy subjects (NTs) were detected by flow cytometry. Expression levels and phosphorylation of Cx43 protein in peripheral blood lymphocytes of EHs and NTs were analyzed by Western blot. The proliferation rate of peripheral blood mononuclear cells (PBMCs) after treatment with a Cxs inhibitor was examined by a CCK-8 assay. The levels of inflammatory cytokines were detected using ELISA. Within the CD3^+^ T cell subsets, we found a significant trend toward an increase in the percentage of CD4^+^ T cells and CD4^+^/CD8^+^ ratio as well as in serum levels of IFN-γ and TNF-ɑ in the peripheral blood of EHs compared with those in NTs. Moreover, the peripheral blood lymphocytes of EH patients exhibited enhanced GJCs formation, increased Cx43 protein level and Cx43 phosphorylation at Ser368, and a significant increase in Cx40/Cx43 surface expressions levels in CD4^+^ or CD8^+^ T lymphocytes. Cx43-based channel inhibition by a mimetic peptide greatly reduced the exchange of dye between lymphocytes, proliferation of stimulated lymphocytes and the pro-inflammatory cytokine levels of EHs and NTs. Our data suggest that Cx40/Cx43-based channels in lymphocytes may be involved in the regulation of T lymphocyte proliferation and the production of pro-inflammatory cytokines, which contribute to the hypertensive inflammatory response.

## Introduction

Hypertension, a worldwide public health problem, is the major risk factor for both cardiovascular- and stroke-associated diseases. Worldwide, hypertension has also become one of the major causes of death and disease burden [1]. Despite the prevalence of essential hypertension, the pathogenesis of this condition is not completely understood. Low-grade inflammation plays a crucial pathogenic role in hypertension. A large body of evidence has suggested that innate and adaptive immune system responses are involved in hypertension-mediated low-grade inflammation [2]. Ang II- and high salt induced hypertension are associated with vascular infiltration of inflammatory cells, including T cells, B cells, monocytes, macrophages and dendritic cells (DCs) [3, 4]. All inflammatory mechanisms, including adhesion molecule and chemokine expression, immune cell activation and cytokine release and oxidative stress, appear to be triggered during hypertension [5]. More recent studies have significantly expanded our understanding of the role of lymphocytes in blood pressure (BP) regulation, especially T cells, to the development and progression of hypertension in hypertensive animal models [3, 6, 7]. T cell-derived cytokines IL-2 and IFN-γ (interferon-gamma), and their expression levels are significantly up-regulated in hypertensive mice [8]. Additionally, the suppression of T cell-driven target organ inflammation ameliorates or prevents experimental hypertension [6, 9, 10]. Essentially, these studies and reviews indicate that T cell-mediated inflammatory responses are necessary for the induction of hypertension.

The homeostasis of the immune system and efficient immune responses against chronic pathologies (e.g., hypertension and diabetes) require efficient coordination between different immune cell types and are controlled by the actions of three communication mechanisms at the intracellular, extracellular and intercellular levels [11]. Communication at the intercellular level is mainly mediated by gap junction channels (GJCs) [11, 12]. Hemichannels, or connexons, are made up by 6 connexin proteins. Two connexons, formed by 6 connexin proteins each in adjacent cell membranes, can form a gap junction channel. GJCs and HCs consist of two protein families: Cxs or pannexins (Panxs), which are present in almost all immune cells [13, 14]. Cxs-based hemichannels (Cx-HCs) are composed of six identical Cxs (homomeric connexon) or a mixture of Cxs types (heteromeric connexon). Compared to the Cxs, native Panxs are similar to Cxs in membrane topology, but they form hemichannels only due to the glycosylation of their extracellular loops [15].

Most immune cells (i.e., T cells, B cells, mast cells, follicular DCs and macrophages) have been found to express Cxs and form homotypic interactions within themselves or heterotypic interactions with other immune cells via GJCs [13, 14]. Immune cells form GJCs in response to inflammatory stimuli, which leads to “rosette” formation [15]. GJCs transfer not only electrical and biochemical signals but also immunorelevant molecules between neighboring cells in the form of ions, second messengers, small metabolites and peptides [13]. Previous research has suggested that GJCs are necessary for the further maturation of T and B cells in the thymus and peripheral lymph nodes [14]; thus, GJCs in immune cells play a pivotal role in specific immune responses. Cx-based HCs also have distinct roles in communication between the cytosol of an individual cell and its extracellular environment and in the transportation of many signaling molecules, including ATP, nicotinamide adenine dinucleotide, glutamate, and prostaglandins [11]. Furthermore, these leukocytes may release ATP via HCs. Recent data have indicated that Cx-based HCs act as modulators of the DC-T cell signaling machinery that modulates T cells activation, and cytokine release [16].

Cxs are encoded by a multi-gene family consisting of 20–21 members in mammals [17]. Of the 21 different human *Cx* genes [17], Cx40 and Cx43 are the most important Cxs proteins in the immune system [18]. More evidence has suggested that Cx43 is involved in the proliferation and differentiation of mature T cells and the secretion and production of cytokines [19]. Activation of CD4^+^ T lymphocytes is associated with the up-regulation of Cx43 expression [20] and CD4^+^ T lymphocytes establishes intercellular communication with macrophages [21]. Cxs and GJCs also participate in Ig secretion and cytokine production, and inhibition of GJCs impairs cytokine release by T cells [22]. Moreover, cytokine modulation of GJCs and HCs leads to a rapid propagation and coordination of activating or inhibitory signals among neighboring immune cells [13]. For example, TNF-α (tumor necrosis factor alpha) plus IFN-γ potentiate Cx43 functional expression and formation of GJCs between human monocytes/macrophages that enhance immune responses [23]. The above mentioned studies indicate that Cxs are important cell surface components that modulate immune processes, and their expression or activity can also be regulated by inflammatory signals.

Although Cxs-based channels have been identified both in vitro and in vivo in T cell, their role in T lymphocytes in the regulation of the hypertensive inflammatory response remains unclear. In the current study, we demonstrate that alteration of peripheral blood T cell subsets of patients with essential hypertension is associated with a significant increase in Cx43 expression and phosphorylation. Moreover, we identify a role for Cx43 formed GJCs in the regulation of T lymphocyte proliferation and pro-inflammatory cytokines release during hypertension.

## Materials and methods

### Human study and ethical approval

All the subjects were from the First Affiliated Hospital of Medical College of Shihezi University in Xinjiang, China. Samples were obtained from 40 essential hypertensive patients (EHs) (average age: 56.14±2.19 years; range of age: 45–65 years, 20 men, 20 women) and 40 normotensive healthy subjects (NTs) (average age: 53.60±3.45 years; age range: 45–65 years, 20 men, 20 women), selected by the case-control study method. All patients underwent subjective examinations in the morning after an overnight fast. BP measurement was performed in the patients’ dominant arm after more than 10 min rest in a seated position. Hypertensive status was determined according to BP >140/90 mmHg. There was no blood relationship between subjects. All patients met the following World Health Organization-International Society of Hypertension criteria: hypertension was defined as systolic BP (SBP) ≥140 mmHg and/or diastolic BP (DBP) ≥ 90 mmHg with repeated measurements or receiving anti-hypertensive medication. Patients with secondary hypertension; endocrine diseases (diabetes); or heart, liver and kidney diseases were excluded by history or physical examination. This study was conducted in accordance with the declaration of Helsinki, with approval from the Institutional Ethics Review Board (IERB) at the First Affiliated Hospital of the Shihezi University School of Medicine (IERB no. SHZ2010LL01). Written informed consent was obtained from all participants.

### Cell culture and intervention treatment assay

Peripheral blood mononuclear cells (PBMCs) from EHs and NTs were harvested by Ficoll-Paque Premium (Cat. No. GE17-5442-02; Sigma Aldrich, St. Louis, Missouri, USA) density gradient centrifugation. PBMCs (1×10^6^/well) were incubated for 2 *h* in serum-free medium at 37°C under an atmosphere with 5% CO_2_. Non-adherent cells were removed, and the remaining cells were incubated in the presence of 1000 U/ml recombinant human IL-2 (ProSpec-Tany TechnoGene Ltd., Rehovot, Israel) and Gap27 [500 µM, a peptide (SRPTEKTIFII) derived from Extracellular loop II of connexin 43] (Cat. No. A1045; ApexBio Technology LCC, Houston, TX, USA). The cells from EHs and NTs were divided into the following groups: PBMCs (positive control); PBMCs+IL-2; PBMCs+IL-2+Gap27. The cultures were maintained for 72 *h*. All cells were cultured for 48 h in RPMI-1640 (Gibco, Carlsbad, CA, USA) with 10% FBS. The same volume of pure RPMI-1640 containing 10% FBS was used as a blank.

### FACS analysis

Whole blood (3 ml) was collected from each subject into sterile tubes with EDTA, and subjected to isolation of PBMCs by using Ficoll-Paque Premium (Sigma Aldrich, St. Louis, Missouri, USA) density gradient centrifugation. Red blood cells in the samples were lysed with 2 ml FACS™ Lysing solution (Cat. No. 349202; Becton Dickinson Immunocytometry Systems, San Jose, CA, USA) for 10 min at room temperature, and then washed twice with PBS. PBMCs were washed once with PBS and adjusted to a density of 2×10^6^ cells/ml in 50 µL PBS, and then PBMCs viability was assessed with 1:1000 LIVE/DEAD® Fixable Aqua amine-reactive dye (Cat. No. L34957; Invitrogen Corporation, Carlsbad, California, USA) for 20 min in the dark at room temperature. Cell-surface staining in T cells was performed by adding 5 µl primary monoclonal antibodies in each tube containing, including isotype control antibody, FITC-conjugated anti-human CD3, PE-conjugated anti-human CD4 and APC-conjugated anti-human CD8 (Cat. No. 400207 for isotype control antibody; Cat. No. 300306 for FITC-conjugated anti- human CD3 antibody; Cat. No. 344606 for PE-conjugated anti-human CD4 antibody; Cat. No. 300912 for APC-conjugated anti-human CD8 antibody; Biolegend, Inc., San Diego, CA, USA), to each tube. All antibodies were prepared according to the manufacturer’s protocols and were incubated with whole blood for 30 min at room temperature in the dark, followed by analysis on a FACSort™ flow cytometer (Becton Dickinson). The lymphocyte population was gated based on their physical features in a region according to their characteristic forward scatter (FCS) and side scatter (SSC) patterns; background staining was reduced by the creation of a dump channel for dead cells (Live/Dead Fixable Aqua Dead Cell Stain Kit, Invitrogen). For the analysis of Cx40 and Cx43 expression levels on the surface of T-lymphocyte subtypes, a 30 min permeabilization was performed with Cytofix/Cytoperm Kit (Cat. No. 554714; BD Biosciences, San Jose, CA) after CD4-PE and CD8-APC primary Ab incubation. After permeabilisation and incubation with unlabeled mouse monoclonal anti-Cx40 (Cat. No. sc-365107; Santa Cruz, CA, USA) or anti-Cx43 antibodies (Cat. No. sc-271837; Santa Cruz, CA, USA) directed to the C-terminal cytoplasmic domain, cells were incubated on ice for 30 min with a FITC-labelled rabbit anti-mouse antibody from Sigma-Aldrich (Sigma-Aldrich, St Louis, MO, USA). The analysis of T-lymphocyte subtypes and Cx40/Cx43 expression using two-color immunofluorescence flow cytometry method using a flow cytometer (FACSort; BD Pharmingen) with BD CellQuest Pro software (version 2.0, system OS2, Becton Dickinson). In addition, for cytokine expression, mononuclear cells were stimulated with 1000 U/ml IL-2, 500 µM Gap27 plus 1000 U/ml IL-2 and were fixed in 2% paraformaldehyde for 15 min. Then, the cells were washed in PBS and immunophenotyping of the samples was performed with CD4-PE and CD8-APC primary Ab. After the staining of surface antigens, intracellular staining was performed using a Cytofix/Cytoperm Kit. Intracellular cytokines were measured using the following different fluorophore-conjugated primary Abs: FITC-conjugated anti-human IFN-γ (Cat. No. 552882; BD Biosciences, San Diego, CA) and Alexa Fluor 488-conjugated anti-human TNF-ɑ (Cat. No. 557722; BD Biosciences, San Diego, CA).

### Calcein-acetomethoxy transfer mediated gap junctional intercellular communication (GJIC) detection by flow cytometry

A gap junction permeant green fluorescent dye, Calcein acetoxymethyl ester (calcein AM) in combination with flow cytometry as described previously [21, 24] was used to assess functionality of GJCs between peripheral blood lymphocytes from EHs and NTs. Briefly, Isolated peripheral blood lymphocytes from EHs and NTs were adjusted to a density of 2×10^6^ cells/ml and were incubated for 30 min at 37°C with 10 mM DiIC_18_ (the plasma membrane red fluorescent dye) (Cat. No. D282; Invitrogen, Karlsruhe, Germany) or 2.5 mM Calcein AM (Cat. No. 3099; Invitrogen, Karlsruhe, Germany) in RPMI-1640 supplemented with 10% FBS, respectively. The DiIC_18_ single-labled or Calcein single-labeled peripheral blood lymphocytes were washed three times with PBS and BSA (1%, w/v) and then cocultured at a ratio of 1: 100 (Calcein loaded: DiIC_18_-loaded) in the presence or absence of IL-2 and Gap27. Cocultured peripheral blood lymphocytes were stimulated with in the absence or presence of IL-2 (1000 U/ml) added to cells 30 min after seeding. The specificity of dye transfer through GJCs was detected in parallel experiments of cocultures using similar conditions evaluated in the presence of 500 µM Gap27. After 3 hour in coculture, Cocultured peripheral blood lymphocytes were harvested by centrifugation at 2500×g for 3 min at room temperature, washed three times in PBS containing 10 mM EDTA and resuspended with a solution of PBS and 1% BSA. Calcein AM (excitation at 488 nm and emission at 535 nm) and DiIC_18_ (excitation at 488 nm and recorded at 585 nm) fluorescence were detected by flow cytometry as described above. To avoid dye leakage due to cell death, a high percentage of peripheral blood lymphocytes viability (above 98%) were used in this experiment, and cell viability was assessed with 1:1000 LIVE/DEAD® Fixable Aqua amine-reactive dye for 20 min in the dark at room temperature. Communication was assessed with the same flow cytometric parameters.

### Western blot

Total protein extraction solution (Cat. No. 78510; Pierce Biotechnology Inc., Rockford, IL, USA) was used to extract the leukocytic total protein of EH and NT patients. The protein concentration was estimated by a BCA protein assay. Each sample with equal amounts of protein (15 μg/lane) was separated by 10% SDS-PAGE electrophoresis. The resolved proteins were then transferred to a PVDF membrane (Millipore, Billerica, MA, USA). The membranes were blocked with 5% non-fat milk in TBST buffer (pH 8.0, 10 mmol/l Tris-HCl, 150 mmol/l NaCl and 0.2% Tween-20) for 1 h at room temperature and then probed with various primary antibodies (anti-Cx43 polyclonal antibody (1:1000) (Cat. No. 3512S/0004), anti-phospho Cx43 (pS368) (1:1000) polyclonal antibody (Cat. No. 3511S/0003) and anti-GAPDH monoclonal antibody (Cat. No. 8/2118S) (1:1000)) (Cell Signaling Technology, Inc., U.S.A.) overnight at 4°C. After the primary antibody incubation, the blots were washed three times (5 min each) with TBST and incubated with secondary antibody (1:10000; horseradish peroxidase-conjugated goat anti-rabbit or goat anti-mouse secondary antibodies (Beijing Fir Jinqiao Biotechnology Company, Beijing, China)) for 1.5 h at room temperature. Next, the blots were washed five times (5 min each) with TBST and visualized on X-ray film using an ECL chemiluminescence reagent (Cat. No. RPN2109; GE Healthcare Life Sciences, Chalfont, UK). The optical density (OD) of each target protein band was assessed with Quantity One software (Bio-Rad, Hercules, CA, USA) and normalized to the density of corresponding GAPDH bands in the same sample.

### Cell proliferation assay

A CCK-8 Kit (Dojindo Molecular Technologies, Inc., Kumamoto, Japan) was used to assess the immunomodulatory effect of Gap27 on PBMCs following stimulation with IL-2. The procedure was carried out according to the manufacturer's instructions. The inhibitory effects of Gap27 on lymphocyte proliferation were evaluated by comparing the OD in cells co-cultured with Gap27 with the OD of lymphocytes cultured without Gap27. The OD value in every culture well was measured by photodensitometry.

### ELISA assay

The IFN-γ and TNF-ɑ levels in the serum and cell cultural supernatants were measured by sandwich enzyme-linked immunosorbent assays (ELISA), following the manufacturer’s instructions (R&D Systems, Minneapolis, MN, USA).

### Statistical analysis

The experimental data are expressed as the mean ± SEM and analyzed by Student’s *t*-test for the comparison of two groups or by one-way analysis of variance (ANOVA). These analyses were followed by a post-hoc test (Dunnett’s T3) or Tukey’s test for the comparison of more than two groups. The Pearson product-moment correlation coefficient (r) was used to assess the relationship between Cx40 and Cx43 surface expression levels and the expression and serum level of pro-inflammatory cytokines. Statistical analysis was performed by SPSS 19.0 software, and the value of *p* < 0.05 was considered statistically significant.

## Results

### Hypertension results in the changes in T lymphocyte subtypes of peripheral blood in EHs

**Fig 1:**
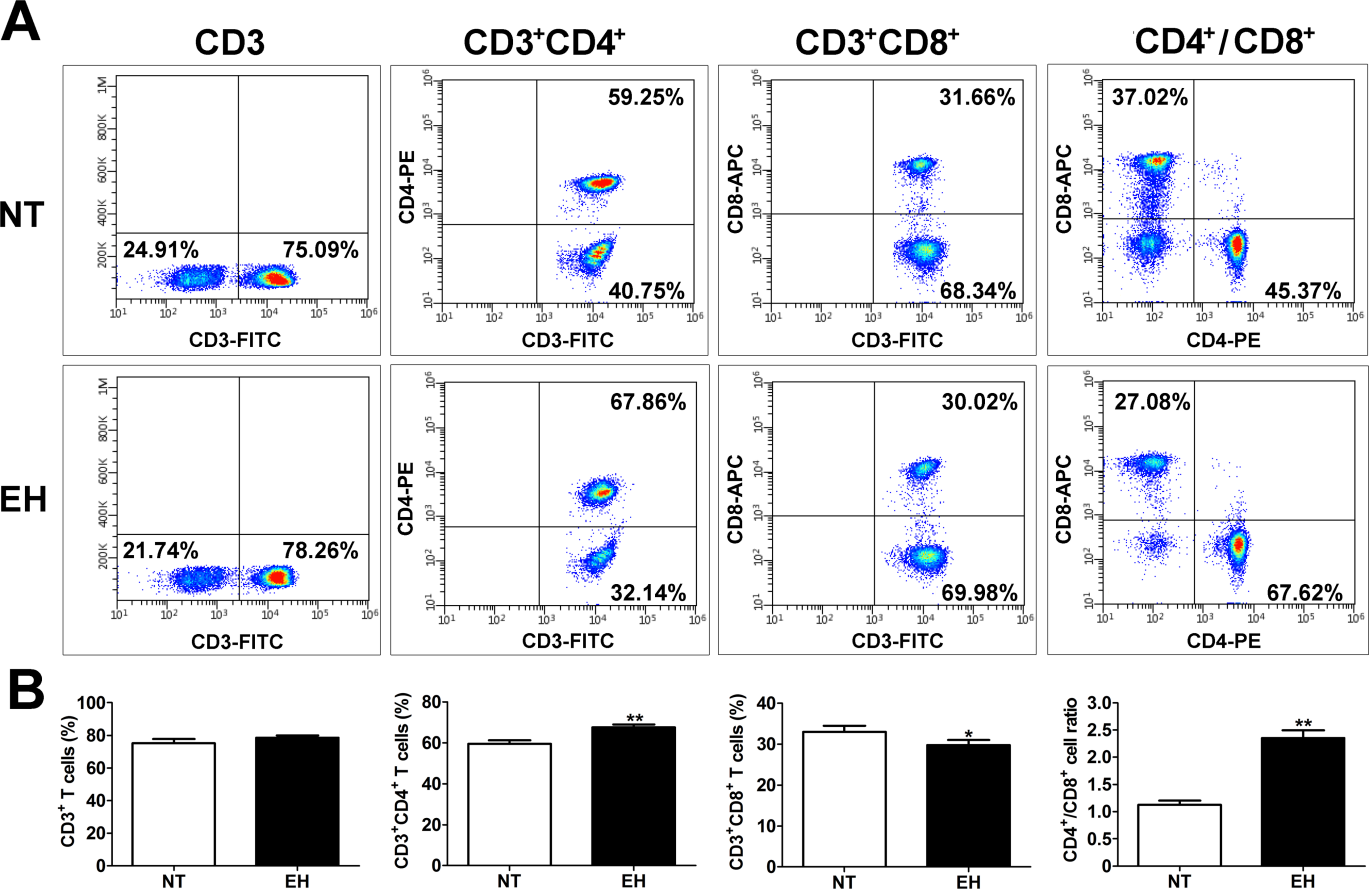
EH induced changes in subtypes of peripheral blood T lymphocytes. A, Representative flow cytometry analysis showing expression levels of circulating subtypes of T lymphocytes in the peripheral blood of 20 EHs and 20 age- and sex-matched NTs. Fresh, resting PBMCs from EHs and NTs were stained with antibodies against CD3, CD4, and CD8 molecules and analyzed by flow cytometry. Based on the CD3^+^ gate, cells were further gated based on CD4 or CD8 expression and the frequency of CD4^+^ or CD8^+^ T cells was determined. B, Frequency of CD3^+^, CD4^+^, and CD8^+^ T cells in the peripheral blood of EHs and NTs. Fresh, resting PBMCs from EHs and NTs were stained with anti-CD3, anti-CD4, and anti-CD8 and then the cells were gated for CD3 positive cells, CD3 and CD4 or CD8 double positive cells after gating for lymphocytes by forward and side scatters. The vertical axis represents the frequency of various T lymphocyte subtypes. Quantitative analysis of the mean percentage of cells ± SEM. *p < 0.05 and **p < 0.01, compared with the NT group (n = 20). EH, essential hypertensive; NT, normotensive.

Several studies have suggested that T cells contribute to BP elevation and vascular dysfunction. The proportions of CD4^+^ and CD8^+^ T cells from EHs and NTs were analyzed by flow cytometry to determine which subtype of T cells contributes to the inflammatory pathogenesis of hypertension. As shown in Fig 1, although there was no difference in the percentage of CD3^+^ T cells between NTs and EHs (*p* > 0.05), a significantly higher percentage of CD3^+^CD4^+^ T cells (*p* < 0.01) and higher ratio of CD4^+^/CD8^+^ (*p* < 0.01) were noted in EHs than those in NTs.

### Hypertension enhances the production of IFN-γ and TNF-ɑ in peripheral CD4^+^ and CD8^+^T cells and serum

**Fig 2:**
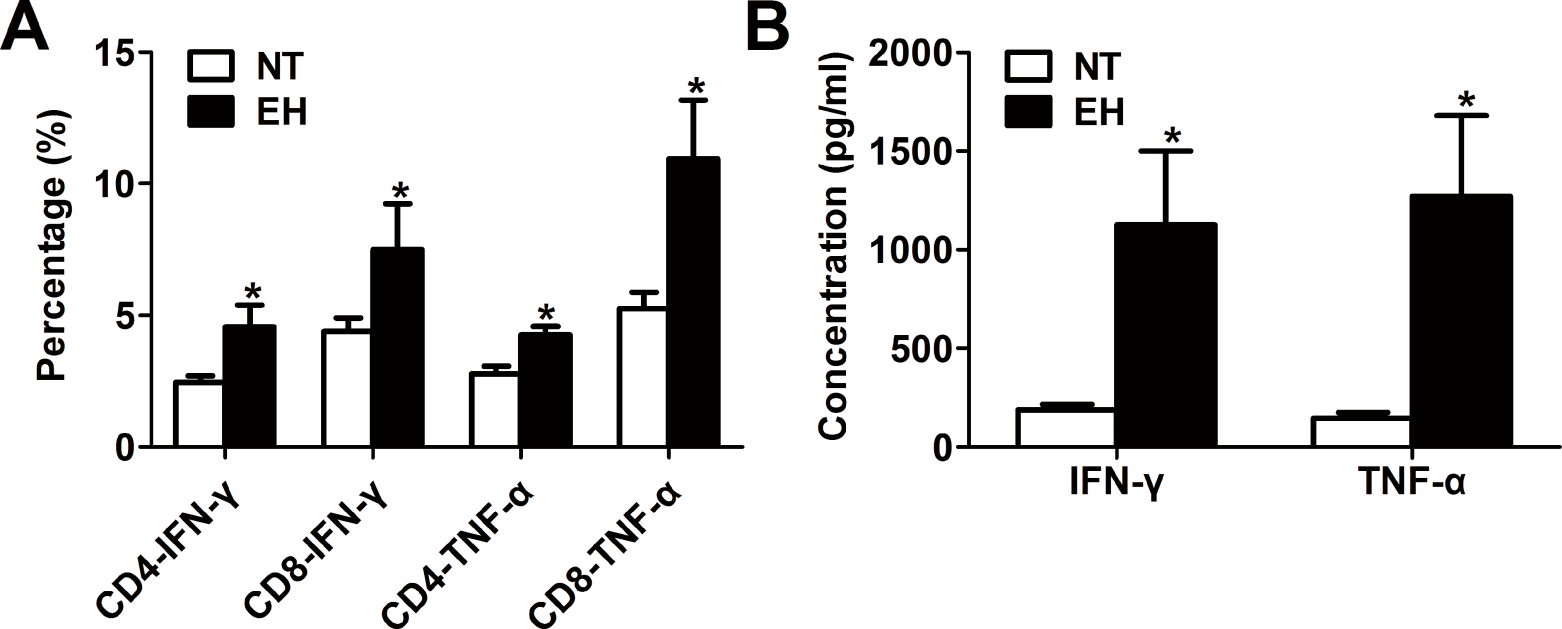
The effect of EH on the expression and production of pro-inflammatory cytokines. A, Quantitative analysis of intracellular staining of pro-inflammatory cytokine (IFN-γ or TNF-α) expression levels in CD4^+^ and CD8^+^ T lymphocyte population of EHs, as determined by fluorescence activated cell sorting analysis. PBMCs from EHs and NTs underwent surface staining with antibodies against CD3, CD4, and CD8 molecules. After surface staining, the cells were fixed and permeabilized and stained with FITC-conjugated anti-human IFN-γ and Alexa Flour 488-conjugated anti-human TNF-ɑ. Based on the CD4^+^ or CD8^+^ gate, cells were further gated based on IFN-γ and TNF-ɑ expression levels, and the percentage of CD4^+^ or CD8^+^ T cells producing IFN-γ and TNF-ɑ was determined. The results shown are the values ± SEM of the percentage IFN-γ- or TNF-α-producing CD4^+^ or CD8^+^ T cells; *p < 0.05, compared with the NT group (n = 20). B, EH increased plasma pro-inflammatory cytokine (IFN-γ and TNF-α) production in the serum; the results shown are the mean ± SEM; *p < 0.05, compared with the NT group (n = 20).

T-cell function in hypertension was assessed by measuring CD4^+^ and CD8^+^ T cells that activated pro-inflammatory cytokine release. As shown in Fig 2, T cell subtypes from EHs, with a high percentage of CD4^+^ T cells, were characterized by higher production of pro-inflammatory cytokines (IFN-γ and TNF-α) (*p* < 0.05) than the T cell subtypes from NTs (see S1 Fig for data) (Fig 2). By contrast, although T cells from EHs had a low percentage of CD8^+^ T cells, the generation of IFN-γ and TNF-α in CD8^+^ T cells was enhanced in EHs compared with that in NTs (see S1 Fig for data) (Fig 2). Moreover, EHs showed significantly higher serum levels of IFN-γ and TNF-α than those in NTs (*p* < 0.05). These data indicate that both CD4^+^ and CD8^+^ T cells may be involved in the development of the inflammatory pathogenesis of hypertension in EHs.

### Increased expression of Cxs, phosphorylation of Cx43 and functionality of GJ in peripheral T cells of EHs are correlated with inflammatory pathogenesis

**Fig 3:**
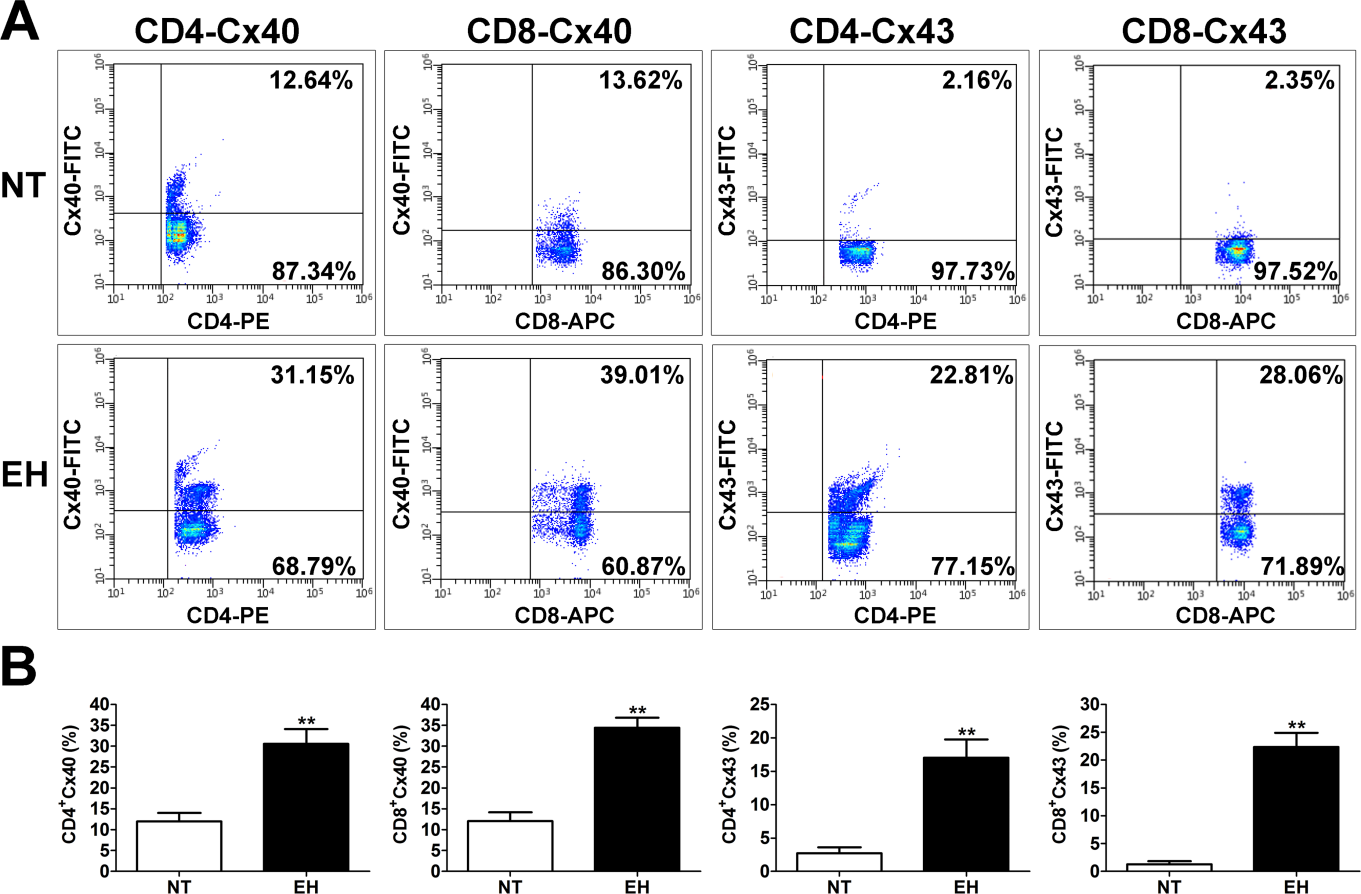
Increased expression of Cxs in CD4^+^ and CD8^+^ T lymphocytes from EHs. A, Representative flow cytometry plots are presented for Cx40 and Cx43 expression levels on gated single-positive CD4^+^ T lymphocytes or CD8^+^ T lymphocyte populations in the peripheral blood from 20 **EHs and 20 age- and sex-matched NTs**. Fresh, resting PBMCs from EHs and NTs underwent surface staining with antibodies against CD3, CD4, and CD8 molecules. After surface staining, the cells were fixed, permeabilized and stained with unlabeled anti–Cx40 or anti–Cx43 plus FITC-labelledsecondary antibodies. Based on the CD4^+^ or CD8^+^ gate, the cells were further gated based on Cx40 and Cx43 expression levels, and the frequency of CD4^+^ or CD8^+^ T cells expressing Cx40 and Cx43 was determined. B, The percentage of CD4^+^ or CD8^+^ T cell population is presented for Cx40 and Cx43. Both Cx40 and Cx43 expression levels are significantly increased in CD4^+^ or CD8^+^ T cells of patients with hypertension compared with those of NTs. Values are mean ± SEM. ** *p* < 0.01, compared with the NT group (n = 20).

We next investigated the surface expression of Cx40 and Cx43 in CD4^+^ and CD8^+^ T cells from the peripheral blood of EHs and NTs. The results showed a significant difference in the expression levels of Cxs in various T lymphocyte subtypes between EHs and NTs (see S2 Fig for data) (Fig 3). Cx40 and Cx43 expression levels were higher in CD4^+^ and CD8^+^ T cells of the EHs (see S2 Fig for data) (*p* < 0.01 and *p* < 0.01, Fig 3) compared with those in NTs.

**Fig 4:**
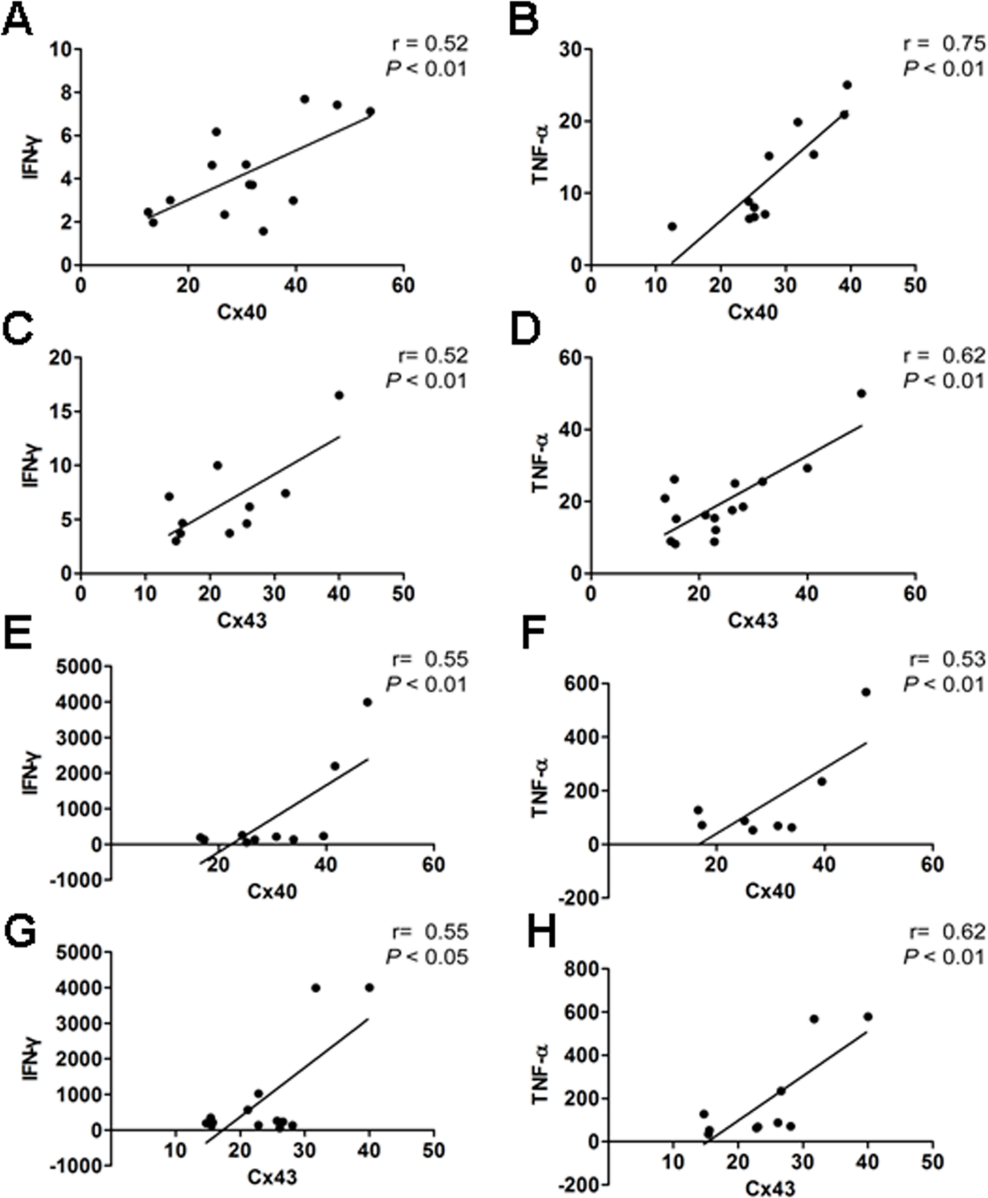
Relationship between T lymphocytes expression levels and serum levels of IFN-γ or TNF-α and Cx40/43 surface expression of T lymphocytes in EHs. Pearson product-moment correlation coefficient (r) test was used to calculate the strength of the correlation. A, The correlation between the expression levels of Cx40 and IFN-γ in the peripheral blood T lymphocytes of EHs. B, The correlation between the expression levels of Cx40 and TNF-α in the peripheral blood T lymphocytes of EHs. C, The correlation between expression levels of Cx43 and IFN-γ in the peripheral blood T lymphocytes of EHs. D, The correlation between the expression levels of Cx43 and TNF-α in the peripheral blood T lymphocytes of EHs. E, The correlation between Cx40 surface expression of peripheral blood T lymphocytes and IFN-γ serum level in EHs. F, The correlation between Cx40 surface expression of peripheral blood T lymphocytes and TNF-α serum level in EHs. G, The correlation between Cx43 surface expression of peripheral blood T lymphocytes and IFN-γ serum level in EHs. H, The correlation between Cx43 surface expression of peripheral blood T lymphocytes and TNF-α serum level in EHs. The results are from two independent experiments.

We extended these results by performing correlation analysis of Cx40 or Cx43 expression and IFN-γ and TNF-α expression and release in EHs. The results revealed an association between the surface expressions of Cx40 and Cx43 in peripheral T lymphocytes and elevated IFN-γ or TNF-α levels (Fig 4). Our results found that T lymphocytes expression levels of IFN-γ was positively correlated with Cxs surface expressions of T lymphocytes in EHs (r = 0.52 for Cx40, r = 0.52 for Cx43, *p* < 0.01, Fig 4A and 4C). Similarly, TNF-α expression levels in T lymphocytes was positively correlated with Cxs surface expression of T lymphocytes in EHs (r = 0.75 for Cx40, r = 0.62 for Cx43, *p* < 0.01, Fig 4B and 4D). The results of the analysis of linear correlation between Cxs surface expressions and serum levels of pro-inflammatory cytokines showed that IFN-γ serum level is positively correlated with Cxs expressions of T lymphocytes in EHs (r = 0.55 for Cx40, r = 0.55 for Cx43, *p* < 0.01 or *p* < 0.05, Fig 4E and 4G). Similarly, TNF-α serum level exhibited positive correlation with Cxs surface expressions in EHs (r = 0.53 for Cx40, r = 0.62 for Cx43, *p* < 0.01, Fig 4F and 4H).

**Fig 5:**
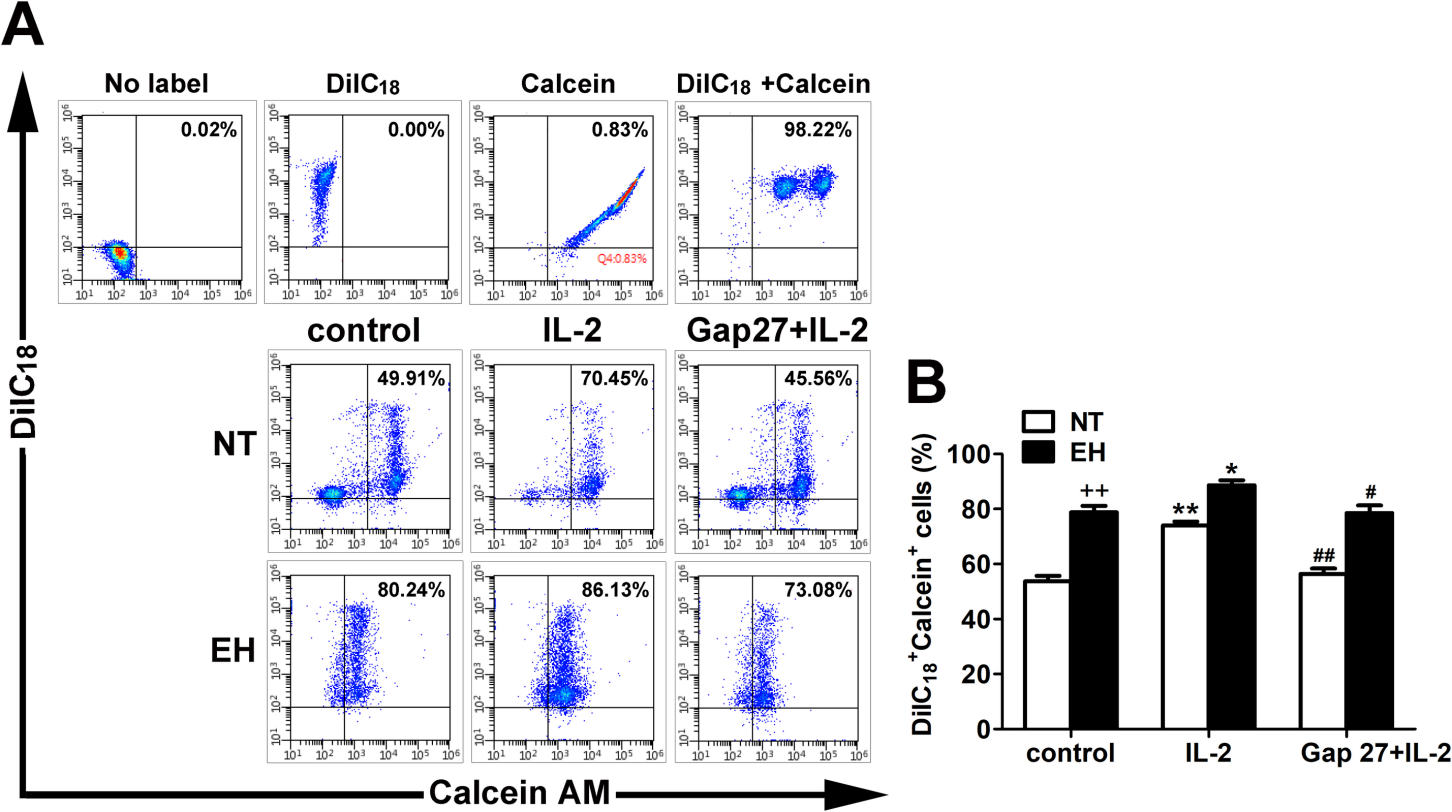
Functional analysis of GIIC between peripheral blood lymphocytes from EHs and NTs. A, Control experiments of donor lymphocytes, recipient lymphocytes and double-labelled fluorescent cells were performed in top parallel; Isolated peripheral blood lymphocytes from EHs and NTs were preloaded with calcein-AM or DiIC_18_, and cocultured for 3 h in absence or presence of IL-2 (1000 U/ml) and Gap27 (500 μM) as described in the materials and methods. Direct calcein transfer through GJCs from donor lymphocytes (single-labelled with calcein-AM) to recipient lymphocytes (single-labelled with DiIC_18_) was assessed by flow cytometry. DiIC_18_-Calcein double-labelled fluorescent cells appear in the upper-right quadrants and expressed as a represent percentage of the total number of peripheral blood lymphocytes in the dot plots. Gap27 was used to assess the specificity of calcein dye transfer. B, The bar graph represents Mean percentages of DiIC_18_-Calcein double-postive cells of three independent experiments ± SEM. ^++^*p* < 0.01 versus NTs control group. *p < 0.05 and **p < 0.01, compared with the control group in the same column (n = 20); #*p* < 0.05 and ## < 0.01, compared with the IL-2-stimulated group in the same column (n = 20).

Intercellular communications mediated by Gap junction between various immune cells has been previously described [21, 24, 25]. To further assess the association between activity of gap junction in adjacent inflammatory cells and abnormality of lymphocytes in proliferation and activation during EH, functionality of GJCs between peripheral blood lymphocytes from EHs and NTs was compared using calcein AM dye transfer assay. Peripheral blood lymphocytes from EHs and NTs were labelled with GJ non-diffusible red fluorescent dye DiIC_18_ and GJ diffusible dye calcein-AM, respectively. After 3 hour in coculture in the absence or presence of IL-2 or Gap27, dye transfer occurred in all groups and that it was higher in peripheral blood lymphocytes stimulated with IL-2 when compared with unstimulated cells (see S3 Fig for data) (*p* < 0.01 in NTs and *p* < 0.05 in EHs, Fig 5A and 5B). There was a significant increase in the amount of calcein-AM dye transfer between cocultured cells in EHs control compared with the transfer from NTs control (see S3 Fig for data) (*p* < 0.01, Fig 5A and 5B). This result is consistent with the enhanced Cx43 expression levels in peripheral blood lymphocytes of EHs. The specificity of the communication through GJCs between peripheral blood lymphocytes was assessed using gap junction inhibitory peptide Gap27. Gap27 showed an obvious inhibiting effect on calcein-AM dye transfer from dye donor cells to recipient cells labelled with DiIC_18_, and the blocking effect was greater in peripheral blood lymphocytes from NTs than that in EHs (see S3 Fig for data) (*p* < 0.01 in NTs and *p* < 0.05 in EHs, Fig 5). These results are similar to data reported previously about the levels of gap junctional intercellular communication (GJIC) blockage by Gap27 in cocultures set up between T or B lymphocytes in the presence of specific antigenic stimulation [25], demonstrating a role for Cx43 in mediating functional GJIC between immune cells. However, we were not able to completely block the dye transfer. The Gap27 only inhibits Cx43 mediated GJIC, and therefore it is conceivable that other Cxs mediated GJIC account for the residual dye transfer.

**Fig 6:**
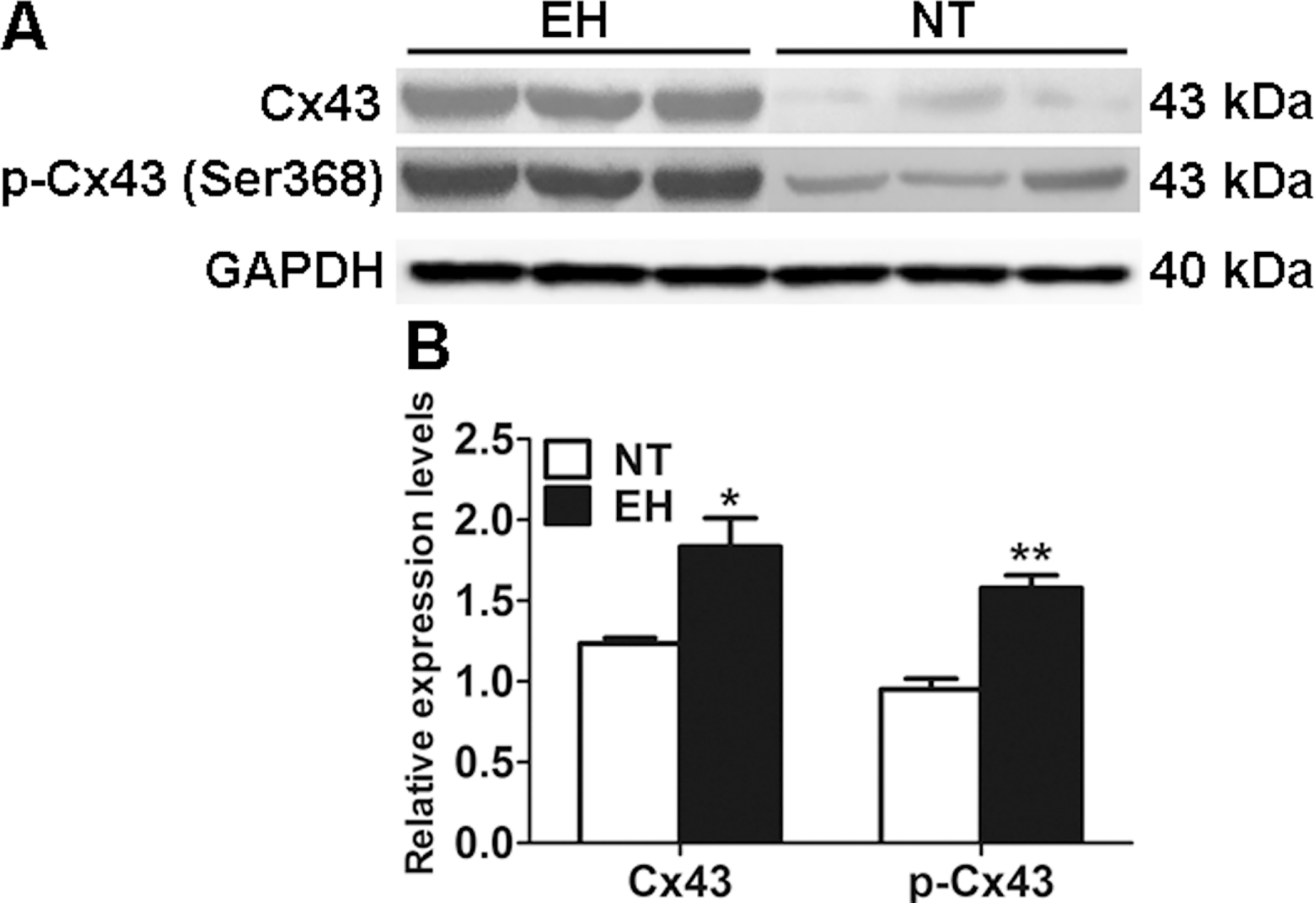
Changes in Cx43 expression and phosphorylation levels in peripheral blood T lymphocytes of EHs. A, Western blots show increased Cx43 phosphorylation and expression levels in EHs. B, The densitometric analysis of Cx43 expression and phosphorylation level in EHs. The data represent the mean ±SEM of three experiments. *p < 0.05 and **p < 0.01 vs. the respective NT control patients; n = 5 per group.

While Cx43 mediated GJCs implied in functionality of immune cells as well as modification of communication between lymphocytes during EH has been demonstrated in the present study, whether this phenomenon is related to expression and phosphorylation of Cxs protein remains unknown. To further confirm whether hypertensive inflammation regulates GJCs by impacting Cx43 protein expression and phosphorylation, we evaluated total Cx43 protein levels and Cx43 phosphorylation levels at p-Ser368 in the peripheral lymphocytes of EHs. As shown in Fig 6, in accordance with the results of surface expression of Cx43 in T cells using flowcytometry, the protein levels of Cx43 (*p* < 0.05, Fig 6A and 6B) and levels of phospho-Cx43 at Ser368 (*p* < 0.01, Fig 6A and 6B) were increased in EHs compared with those in NTs.

### Gap junctions may regulate T lymphocyte proliferation and release of pro-inflammatory cytokines in essential hypertension

**Fig 7:**
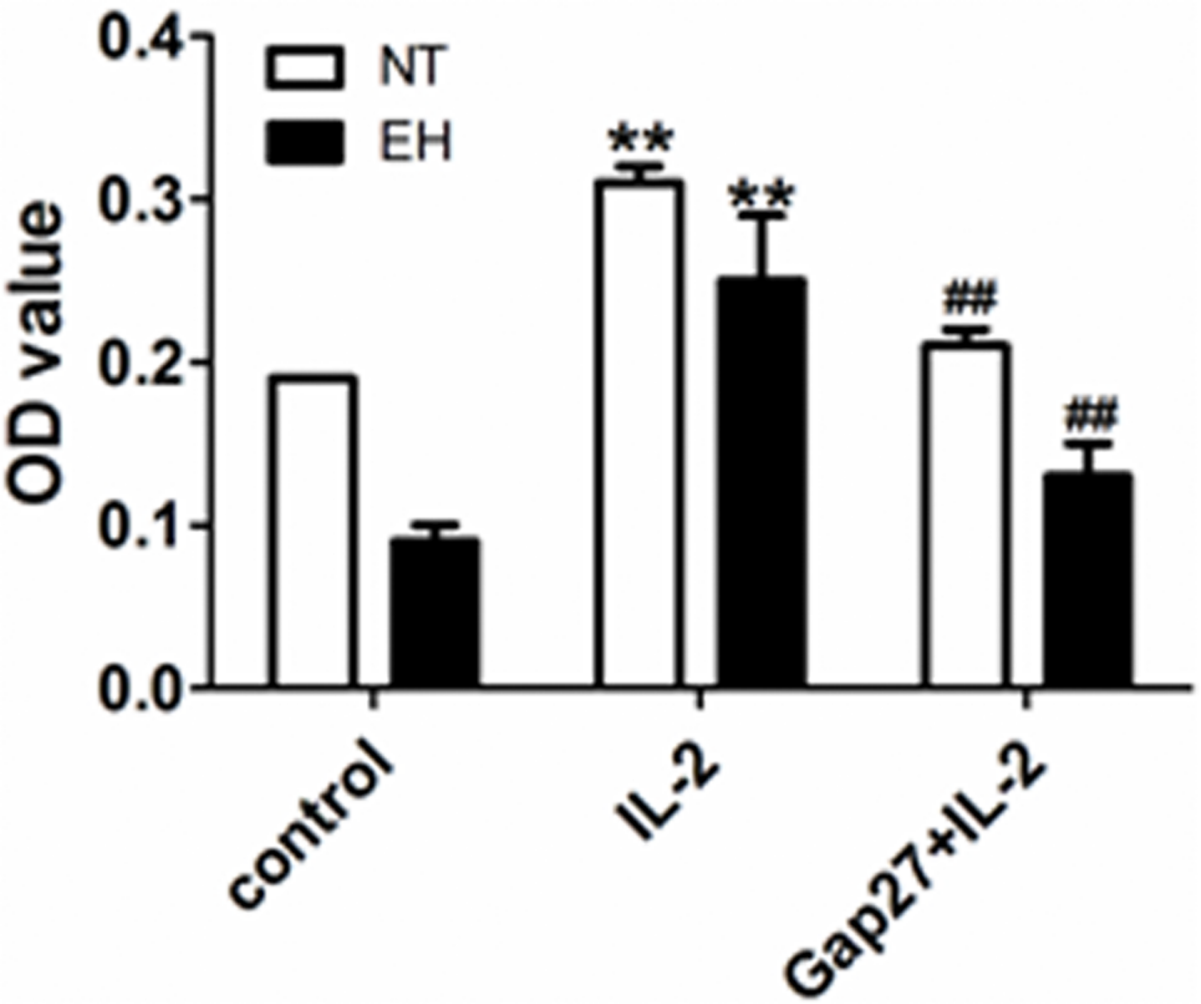
The effect of Gap27 on the proliferation of IL-2-stimulated T lymphocytes from EHs and NTs. Proliferation assay of PBMCs of EHs and NTs stimulated with IL-2 and Gap27, a Cx mimetic peptide. The cells were cultured for 72 h, and cell proliferation was measured with a CCK-8 incorporation assay. Values are the mean ± SEM obtained from three independent experiments. *p < 0.05 and **p < 0.01, compared with the control group in the same column (n = 20); #p < 0.05 and ## < 0.01, compared with the IL-2-stimulated group in the same column (n = 20).

**Fig 8:**
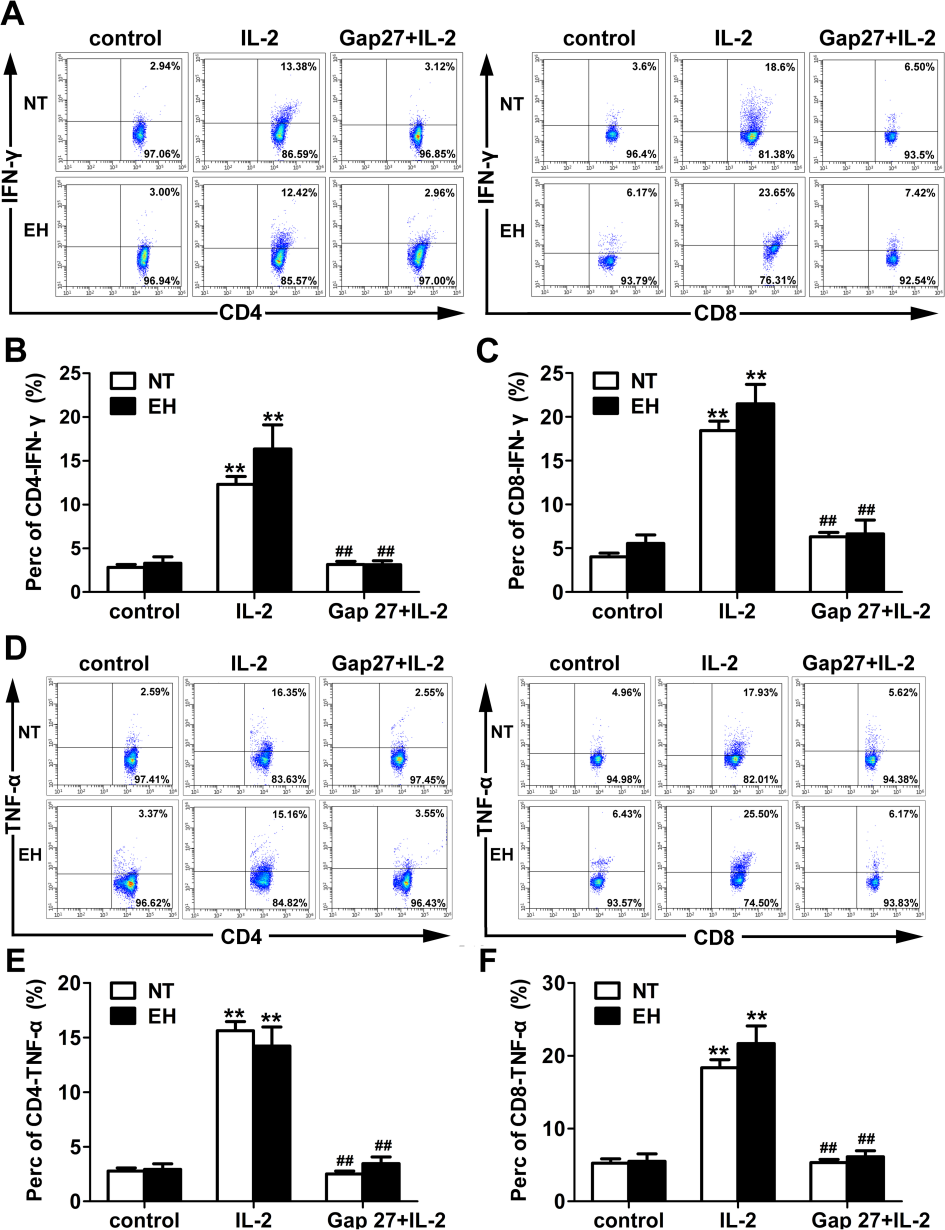
Gap27 alters pro-inflammatory cytokines production in T lymphocytes from EHs and NTs. Representative dot plots of T lymphocytes from unstimulated PBMCs of EHs and NTs. PBMCs of EHs and NTs were stimulated in vitro with IL-2 (1000 U/ml) for 24 h. Gap27 (500 μM) was added to culture supernatants for the last 48 h. Subsequently, the cells were harvested and stained for CD4^+^/CD8^+^, IFN-γ and TNF-α for flow cytometry analysis (A and D). B and C, The percentage of CD4^+^ (B) or CD8^+^ (C) T cells expressing IFN-γ are presented as the percentages of total CD3^+^ T cells. E and F, Percentage of CD4^+^ or CD8^+^ T cells among CD3^+^ T cells expressing TNF-α. Data show the mean ± SEM of the percentage of IFNγ or TNF-α producing CD4^+^ or CD8^+^ T cells. **p < 0.01, compared with the unstimulated group in the same column (n = 20); ^##^*p* < 0.01, compared with IL-2 stimulated group in the same column (n = 20).

**Fig 9:**
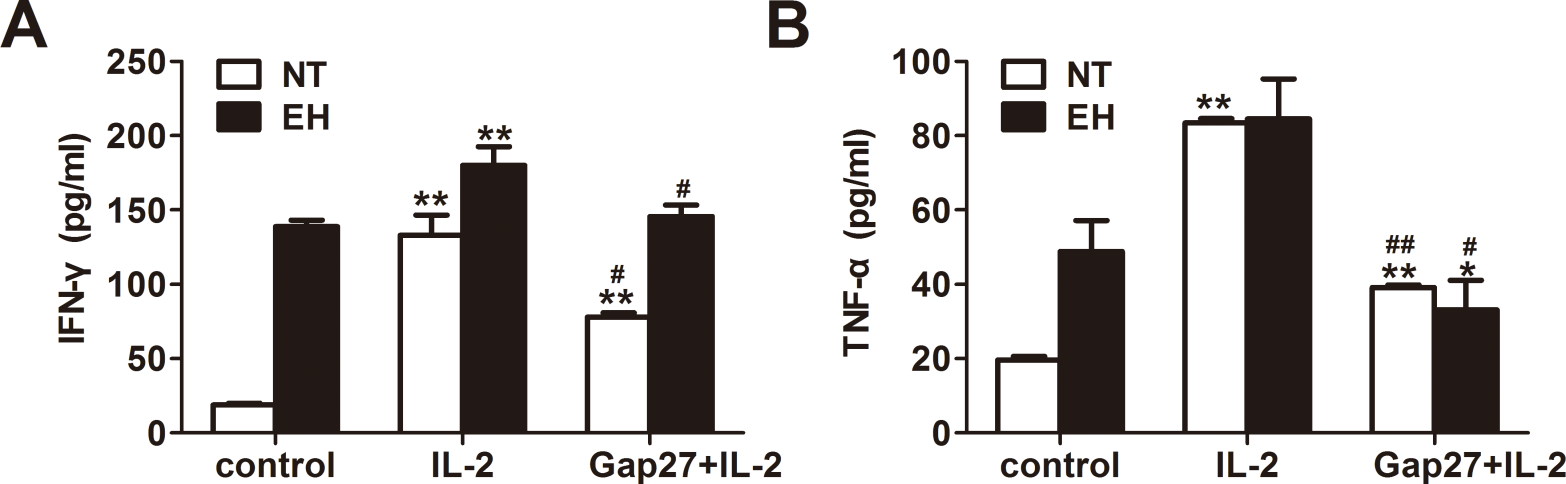
Gap27 inhibits the production of IFN-γ and TNF-α from unstimulated or IL-2-stimulated PBMCs of EHs and NTs. PBMCs from EHs and NTs were cultured in vitro alone or with Gap27 (500 μM) for 48 h in IL-2 (1000 U/ml) culture medium. Supernatants then were analyzed for IFN-γ and TNF-α by ELISA. A, The concentration of IFN-γ in the cell supernatants. B, The concentration of TNF-α in the cell supernatant. Values are the mean ± SEM from three independent experiments. *p < 0.05 and **p < 0.01, compared with the control group in the same column (n = 20); ^#^*p* < 0.05 and ^##^*p* < 0.01, compared with the IL-2-stimulated group in the same column (n = 20).

Blocking Cxs-based channels in lymphocytes results in the suppression of the synthesis and release of pro-inflammatory cytokines, such as IFN-γ and IL-2 [26]. To further investigate the role of Cx40- and Cx43-based channels in T cell proliferation and pro-inflammatory cytokine production during EH, we stimulated cultured peripheral blood lymphocytes from EHs and NTs with IL-2 for 24 *h* or IL-2 for 24 *h* after treatment with the Cx43 mimetic peptide Gap27 for 48 *h*. The results showed that the OD values of T lymphocytes treated with Gap27 plus IL-2 were significantly reduced compared with those of the single stimulation of IL-2 in both EHs and NTs (*p* < 0.01, Fig 7). Similarly, IFN-γ (*p* < 0.01, Fig 8) and TNF-α expressions in stimulated CD4^+^ and CD8^+^ lymphocytes (*p* < 0.01, Fig 8) and their secretion in the culture media (*p* < 0.05, Fig 9) were markedly suppressed by pretreatment with Gap27 in both EHs and NTs. Thus, these data indicate that the induction of T lymphocytes and the synthesis and secretion of pro-inflammatory cytokines maybe directly regulated by Cxs-based channels during essential hypertension.

## Discussion

Although low-grade systemic inflammation has been confirmed during hypertension, as depicted by infiltration of immune cells and elevation of pro-inflammatory cytokine secretion in the serum [2, 5], previous studies have not investigated the roles of Cxs-based channels in the changes in T cell subtypes and increasing pro-inflammatory cytokines production in hypertension. Therefore, the current study aimed to further elucidate the link between gap junctional intercellular communication (GJIC) and inflammation in hypertension.

In the current study, we found the following results: (i) The percentage of CD4^+^ T cells and the CD4^+^ /CD8^+^ ratio significantly increased, and the percentage of CD8^+^ T cells decreased; additionally, the increased synthesis of pro-inflammatory cytokines(IFN-γ and TNF-α) in peripheral blood was associated with hypertensive inflammation. (ii) Increased inflammation during EH resulted in increased Cx40/Cx43 expression levels in CD4^+^ and CD8^+^ T cells, increased Cx43 protein levels, enhanced Cx43 mediated GJIC and Cx43 phosphorylation in peripheral blood lymphocytes. (iii) Inhibition of Cx43 mediated GJCs or HCs of peripheral blood T lymphocytes by Gap27 reduced the synthesis and secretion of EH-driven pro-inflammatory cytokines (IFN-γ and TNF-α). These data demonstrate that Cxs-based channels in T cells may modulate the hypertension-induced inflammatory response.

Recent studies have shown that hypertension causes the activation of T lymphocytes and target-organ damage induced by the infiltration of activated T cells [2, 27]. In particular, CD4^+^ and CD8^+^ T cells are thought to participate in BP control [6], and increasing BP is accompanied by the extensive infiltration of these T cells into key target organs (heart, kidney and vasculature) [2, 6]. Experimental studies have demonstrated that Ang II and DOCA-salt significantly increase vascular infiltration of CD4^+^ and CD8^+^ T cells in male animals [6]. Similarly, adoptive transfer of CD4^+^ T cells from rats with preeclampsia to normotensive pregnant rats led to BP elevation in the recipient rats [28]. This evidence demonstrates that CD4^+^ T cells are the main adaptive immune players in the pathological development of hypertension. In our study, we confirmed the immunologic characterization of peripheral blood T cells from EHs to determine which subtypes of T cells are involved in the elevation of BP. The data revealed that CD4^+^ T cells and the ratio of CD4^+^/CD8^+^ T lymphocytes in peripheral blood were higher in EHs compared with those in NTs (Fig 1). Consistent with previous reports, our data also strongly suggest that hypertensive inflammation is associated with CD4^+^ T cell irregularities in EH development. Moreover, clinical studies have shown that hypertensive patients exhibit an increased percentage of CD3^+^ and CD8^+^ cells in response to acute mental stress compared with that in control groups [27]. However, our results showed that the percentage of CD8^+^ T cells in the peripheral blood of EHs was reduced (Fig 1), which may be caused by enhanced infiltration of CD8^+^ T cells into other tissues [29]. Similar to the increases in the ratio of CD4^+^/CD8^+^ in the kidney of male spontaneously hypertensive rats [30], our data showed that the CD4^+^/CD8^+^ ratio in the peripheral blood of EHs was higher than that in NTs. Unfortunately, we did not use additional cell surface markers to examine the changes in other subpopulations of T cells according to inflammatory cytokine released, including T regulatory cells, NK T cells, and CD4^+^ T cells with Th1 and Th2 phenotypes. These subpopulations of T cells may regulate BP by influencing the kidney and blood vessels remodeling via cytokine release.

In general, activated CD4^+^ T cells, which are induced by different cytokines can differentiate into three types of T cell with different phenotypes, including a T helper-1 (Th1), a Th2 and a Th17 cells [31]. Th1 -polarized cells generate pro-inflammatory cytokines such as TNF-α, IL-2 and IFN-γ [31]. Some evidence suggests that cytokine levels are increased in human hypertension, and serum levels of inflammatory cytokines (IFN-γ, IL-6, and TNF-α) have been positively correlated with BP in humans; thus, they can act as the important biomarkers of low-grade systemic inflammation [32]. These pro-inflammatory cytokines promote vasoconstriction and sodium retention, leading to BP elevation and vascular remodeling. In the present study, compared with NTs, EHs had higher serum levels of IFN-γ and TNF-α (Fig 2). The expression levels of inflammatory cytokines IFN-γ and TNF-α in CD4^+^ and CD8^+^ T lymphocytes were significantly up-regulated in EHs. Indeed, previous research has shown that TNF-α and IFN-γ are produced in excess in hypertensive animal models, contributing to the inflammatory infiltration of blood vessels and exacerbation of hypertension [33]. However, the detailed roles of these cytokines during hypertensive inflammatory responses need to be fully defined.

While a number of studies and our work have shown that the disorder of lymphocyte subtypes plays an essential role in hypertension, the precise mechanisms underlying this role remain unclear. Therefore, comprehending how these lymphocytes are activated and participate in the hypertension-mediated inflammatory response is crucial. Over the past decade, studies have provided compelling evidence that Cxs-based channels, including GJCs and HCs, play an indispensable role in modulating key immunological responses. Cx40 and Cx43 are the main Cxs in T, B and NK cells, with the predominant expression of Cx43 in circulating lymphocytes and Cx40 in lymphocytes derived from secondary lymphoid organs [18]. PHA- and LPS-stimulated T lymphocytes exhibit increased levels of Cx40 and Cx43 proteins and Cxs surface expression in CD4^+^ cells [25]. Recent studies have demonstrated that activation of CD4^+^ T lymphocytes is associated with the up-regulation of Cx43-based GJCs and HCs expression levels and that HCs are of significance for activated T lymphocytes proliferation [20, 34]. However, it is uncertain whether hypertension-mediated inflammatory response induces Cxs expression in T lymphocytes, and whether Cxs are also implicated in hypertensive inflammation induced alterations in T cell proliferation. Excitingly, our results also show that Cx40 and Cx43 expression levels in the membranes of CD4^+^/CD8^+^ T cells were significantly increased in EHs (Fig 3); in particular, the expression of Cx43 in T lymphocytes was strongly up-regulated by the hypertension mediated inflammatory response. Thus, our findings are consistent with the above mentioned studies, suggesting a possible contribution of Cxs, particularly Cx43, to T lymphocyte proliferation during development and progression of hypertension in EHs. Moreover, we found a strong correlation between pro-inflammatory cytokine (IFN-γ and TNF-α) levels (Fig 2) and the expression levels of Cx40 or Cx43 (Figs 3 and 4) in peripheral blood T lymphocytes and the serum of EHs. These results provide an explanation for the importance of pro-inflammatory cytokines in the maintenance of Cxs expression. Different studies have demonstrated that the expression of Cx43 can be induced by pro-inflammatory cytokine in monocytes/macrophages and DCs [23, 35]. IFN-*γ* in combination with TNF-*α* and IL-1*β* increase the synergic response of Cx43 and Cx45 levels in DCs, but treatment with IFN-*γ* alone does not induce Cx43 expression [35]. Therefore, we speculate that increased level of TNF-*α* and IFN-*γ* in T cells and the serum of EHs might modulate the expression levels and functionality of Cx43-based GJCs and HCs in T cells, as it occurs in other immune cells.

Cx40 and Cx43 mediated intercellular communication has been reported to be directly implied in intercellular communication between T, B and NK lymphocytes derived from human peripheral blood, and analysis of dye transfer indicated that they could assemble into functional gap junction channels [24, 25]. Moreover, other immune cells including DCs and macrophages, as well as endothelial cells can communicate with lymphocytes through GJCs [13, 36], and Cx-HCs have been demonstrated in some of them. Similarly, we also detected dye coupling between dye-loaded lymphocytes and unlabelled lymphocytes from EHs and NTs in vitro. Moreover, an important implication of our data is obvious enhanced GJIC in lymphocytes from EHs may be involved in activation of lymphocytes and hypertensive inflammatory response originated from other hypertension mediated inflammatory stimulators. Previous studies has also shown that the functionality of GJIC was regulated by inflammatory stimulators such as LPS and PHA [13]. In contrast to previous report, our results also showed promoting effect of IL-2 on calcein dye transfer between peripheral blood lymphocytes (Fig 5). In the presence of a Cx43 mimetic peptide Gap27, we observed decreased dye coupling from dye donor lymphocytes to receptor lymphocytes, which further supports the hypothesis that Cx43-mediated functional coupling between lymphocytes indicates a direct correlation between hypertensive inflammation or other inflammatory stimulus and modification of direct lymphocyte intercellular communication through GJCs. The inhibitory effect is similar to data reported previously about GJIC blockage by Gap27 in cocultures set up between T lymphocytes [25]. The inhibition was not complete may be result from high connexin membrane turnover under inflammatory stimulus by IL-2. Another reason for the lack of complete inhibition of gap junction coupling is that Gap27 may not block efficiently heterotypic junctions constructed of Cx40/Cx43 [36].

The activity of gap junctions in all types of tissue is known to be highly regulated by different post-translational mechanisms. Undoubtedly, phopshorylation is the main post-translational modification regulating the activities of GJCs or HCs, which have been extensively studied [34]. Phosphorylation of Cx43 at the Ser368 residue is known to decrease the intercellular communication properties of Cx43-based GJCs and is associated with internalization of the protein [37]. Cx43-based GJCs internalization might cause GJCs to undock, and thereby lead to the formation of HCs [38]. In the present study, we observed enhanced phosphorylation of Cx43 at Ser368 in hypertensive patients (Fig 6A and 6B). Oviedo-Orta et al. observed similar results for CD4^+^ T cells. In their study, CD3/CD28 antibodies stimulated CD4^+^ T cells exhibited increased Ser368 phosphorylation of Cx43 compared with unstimulated T cells [20]. However, whether increased Ser368 phosphorylation induce T cell proliferation and other pro-inflammatory events remains unknown. Furthermore, the functional alteration of Cx43-based channels depend on the interplay between different phosphorylation sites.

A possible link between Cx43 and proliferation of T lymphocytes and the release of pro-inflammatory cytokines was suggested by Oviedo-Orta et al.; they showed that prevention of inter-lymphocyte gap junctional communication using Gap27 inhibited proliferation of CD4^+^ T cells in a dose-dependent manner [20]. Mendoza-Naranjo et al. and Tittarelli et al. also demonstrated that Cx43 participates in the regulation of IFN-γ secretion as blocking of Cx43-GJCs remarkably diminished cytokines (IFN-γ, IL-2 and IL-10) secretion by T cells or mDCs and NK cells and thereby inhibited the inflammatory response [16, 21, 24]. These findings demonstrate a role for GJIC in the immune system. To further confirm the specific involvement of Cx43-based channels in the regulation of T cell proliferation and secretion of pro-inflammatory cytokines in EHs, we used the specific inhibitor of Cx43-based channels, Gap 27. Our results indicate that Gap 27 can inhibit the proliferation of stimulated T cells (Fig 7). Production of IFN-γ and TNF-α in the culture media and expression levels in IL-2 stimulated CD4^+^ and CD8^+^ T lymphocytes from patients with EH and NTs were reduced following Gap27 incubation (Figs 8 and 9). This finding suggests that Cx43-based channels may control IFN-γ and TNF-α secretion. In addition, IFN-γ can up-regulate the surface expression of Cx and enhance GJIC in cells of the immune system [16]. This process may result in sustained GJIC between T cells, allowing T cell activation. Although Gap27 has been reported inhibit Cx43-mediated intercellular communication, later studies have found that Gap27 also block Panx1-based and Cxs-based HCs. Compared with GJCs, Cxs-based and Panx1-based HCs are more sensitive to most Cx mimetic peptides [39, 40]. The effect of Gap27 on the conductance of Panx membrane channels was suggested by Wang et al. [39]; the authors showed that Gap27 blocked Panxs-based channel currents in oocytes. Cxs-based and Panx1-based HCs allow the passage of molecules less than 1 kDa such as ATP, between the intracellular space of an individual cell and the extracellular environment [15]. ATP release by Cx-based HCs is a key step that results in immune cell proliferation, the production of pro-inflammatory cytokines and the perpetuation of the inflammasome cycle [11]. Release of ATP from Cx37-based HCs in primary monocytes/macrophages can prevent leukocyte adhesion, which blocks atherosclerotic-mediated inflammation [41]. Several studies have also demonstrated a role of Panxs-based HCs, particularly in ATP release during inflammatory conditions [42]. However, the kinetics of Gap27 inhibition of GJCs and HCs are different, with HCs inhibition occurring more rapidly (minutes) than GJCs inhibition (tens of minutes to hours) [43]. Based on this observation, the incubation time of Gap27 is long enough to completely block the Cx43-based GJC mediated inflammatory response in present study, but whether Cxs-based and Panx1-based HCs are involved in the proliferation of T lymphocytes and the production of pro-inflammatory cytokines in hypertension is unknown. Based on the present data and previous studies, we hypothesize that even though Gap27 inhibits coupling of GJCs and prevents assembly of functional GJCs, the involvement of Cxs-based and Panx1-based HCs cannot be excluded, as mimetic peptides may also bind to Cxs-based and Panx1-based HCs and restrict their role in T cells.

We have shown that the proliferation and activation of T lymphocytes and the production of pro-inflammatory cytokines may be regulated by the expression and functionality of Cxs-based channels; however, the specific channels (GJCs and HCs) involved in the transmission of signaling molecules in vivo that lead to hypertensive inflammation remain unknown. Since that HCs also affect the pro-inflammatory cytokine secretion and proliferation of T cells, we cannot exclude a possible contribution of HCs to these processes in hypertension. Therefore, further studies using a specific mimetic peptide of HCs (Gap19) in combination with conditional knockout mice for Cxs may discern the contribution of HCs to the function and proliferation of T cells under hypertensive conditions.

## Conclusion

In summary, the present results illustrate the importance of GJCs between lymphocytes in immune-mediated hypertensive inflammation. By comparing NTs and EHs, we speculate that Cx40 and Cx43 are involved in the regulation of T lymphocyte proliferation and production of pro-inflammatory cytokines through establishment of GJIC during EH. This link might explain the role of Cxs that leads to the increase in T lymphocyte activation and vascular inflammation in the development of hypertension.

## Supporting information

S1 Fig Flow cytometry analysis of different T-lymphocyte subsets expressing different cytokines in healthy subjects (NTs) and essential hypertensive patients (EHs). (DOCX)

S2 Fig Flow cytometry analysis of Cxs in different T-lymphocyte subsets of healthy subjects (NTs) and essential hypertensive patients (EHs). (DOCX)

S3 Fig Functional experiment of gap junction from peripheral blood lymphocytes of healthy subjects (NTs) and essential hypertensive patients (EHs). (DOCX)
