# Supplementary material for: Up-regulation of gap junction in peripheral blood T lymphocytes contributes to the inflammatory response in essential hypertension
Source: PLoS One. 2017 Sep 14;12(9):e0184773. doi: 10.1371/journal.pone.0184773 (PMC5599050; doi:10.1371/journal.pone.0184773)

**S1 Fig. Flow cytometry analysis of different T-lymphocyte subsets expressing different cytokines in healthy subjects (NTs) and essential hypertensive patients (EHs).**

CD4-IFN-γ and CD8-IFN-γ expression levels of Healthy subjects (NTs)

NT-1: CD4-IFN-γ and CD8-IFN-γ


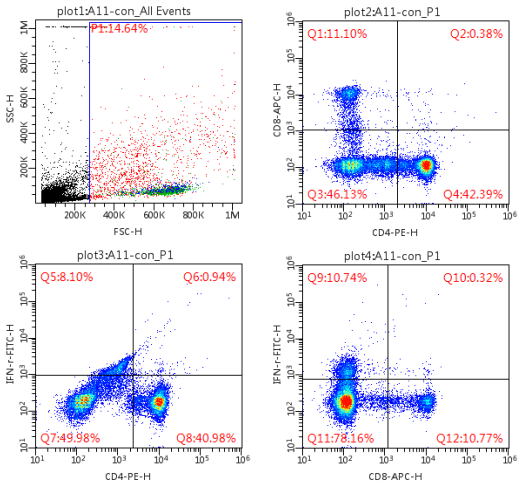


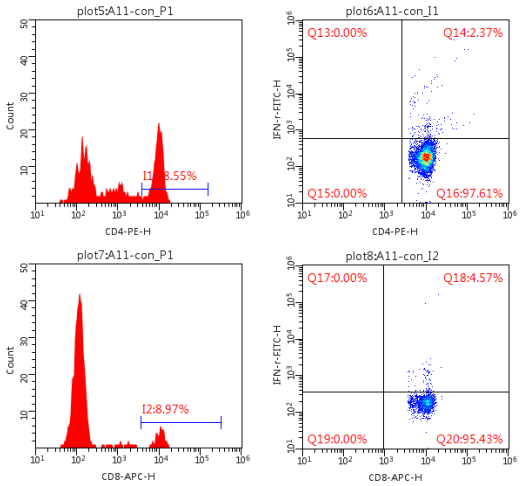


NT-2: CD4-IFN-γ and CD8-IFN-γ


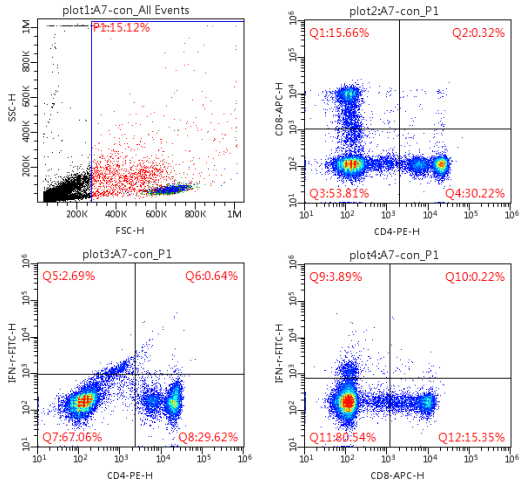


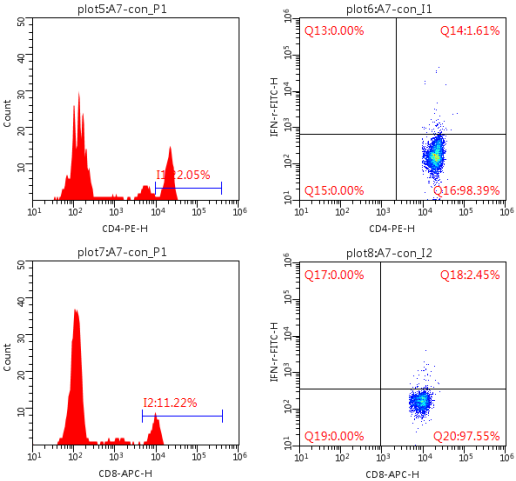


NT-3: CD4-IFN-γ and CD8-IFN-γ


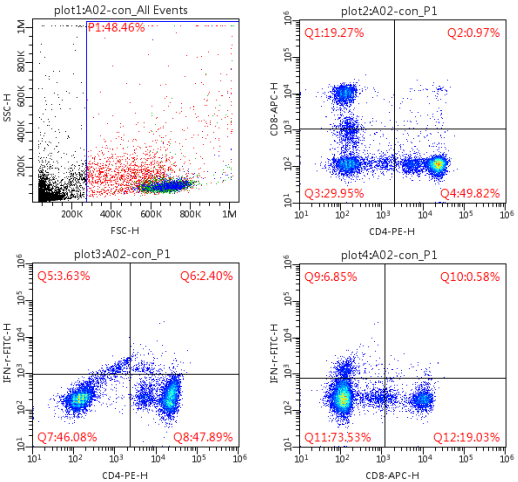


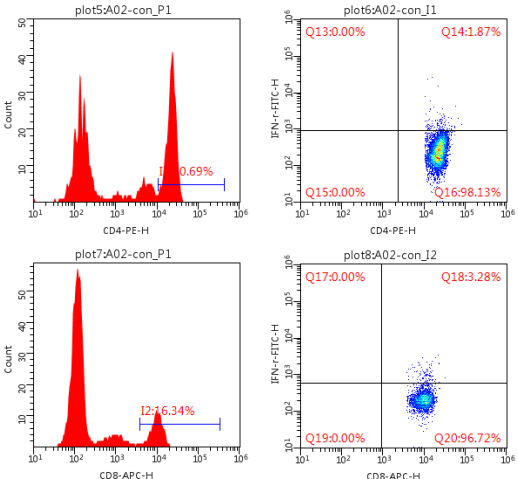


NT-4: CD4-IFN-γ and CD8-IFN-γ


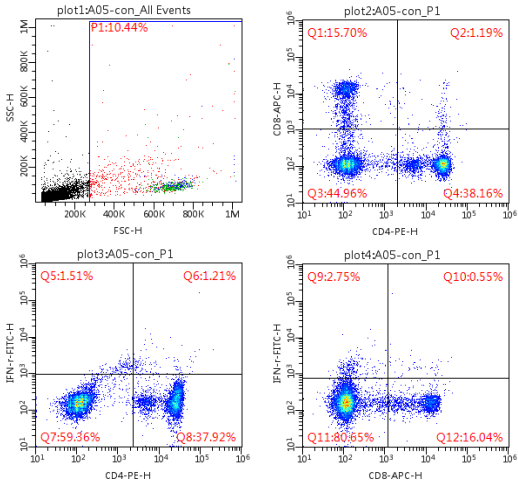


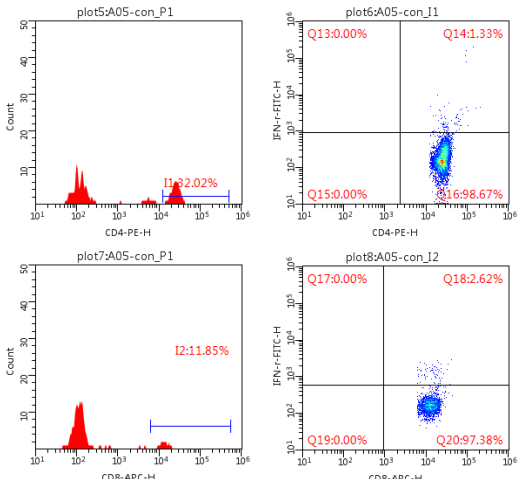


CD4-IFN-γ and CD8-IFN-γ expression levels of Essential hypertensive patients (EHs)

EH-1: CD4-IFN-γ and CD8-IFN-γ


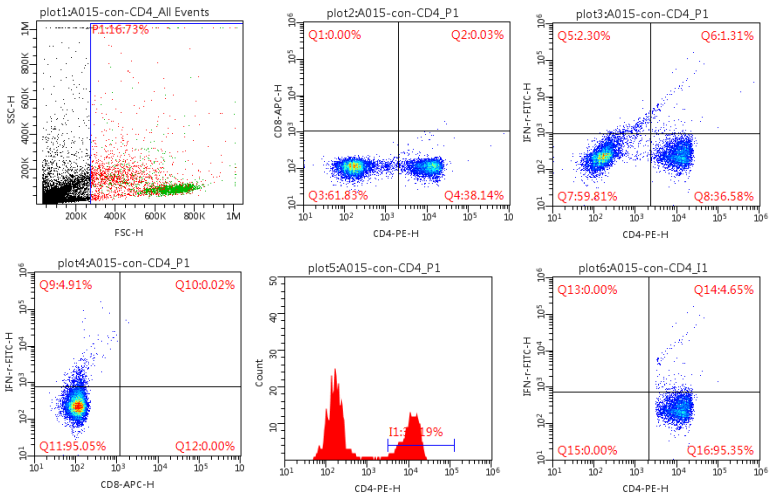


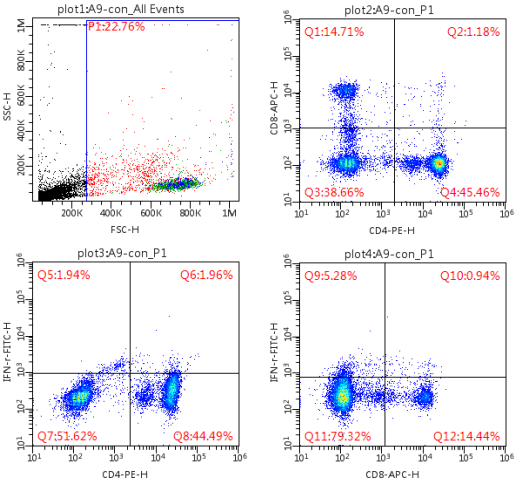


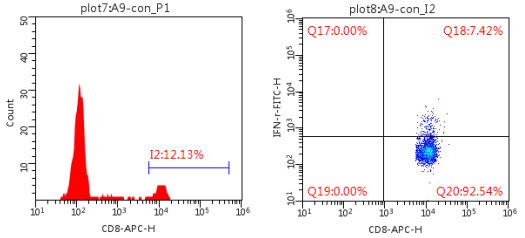


EH-2: CD4-IFN-γ and CD8-IFN-γ


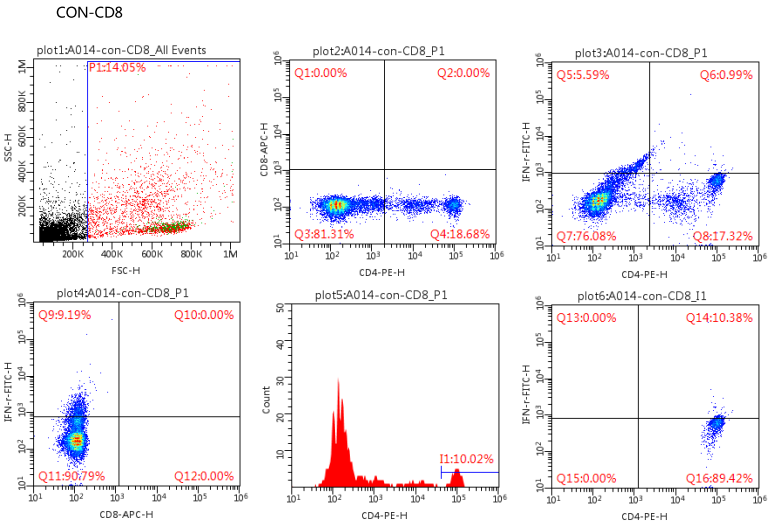


EH-3: CD4-IFN-γ and CD8-IFN-γ


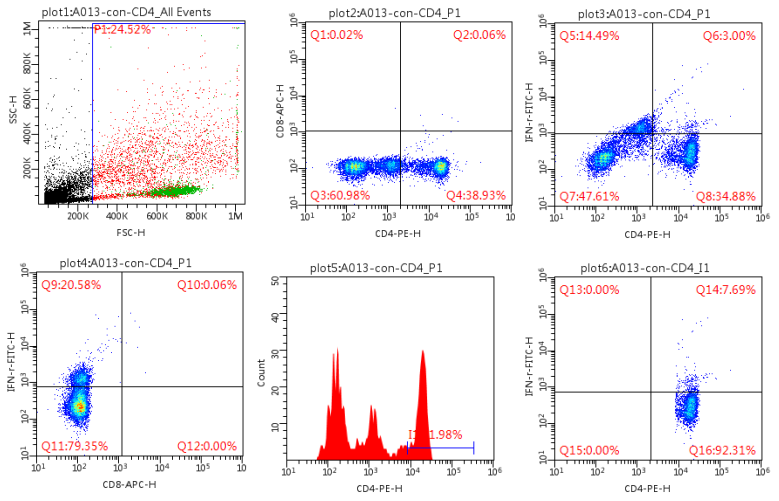


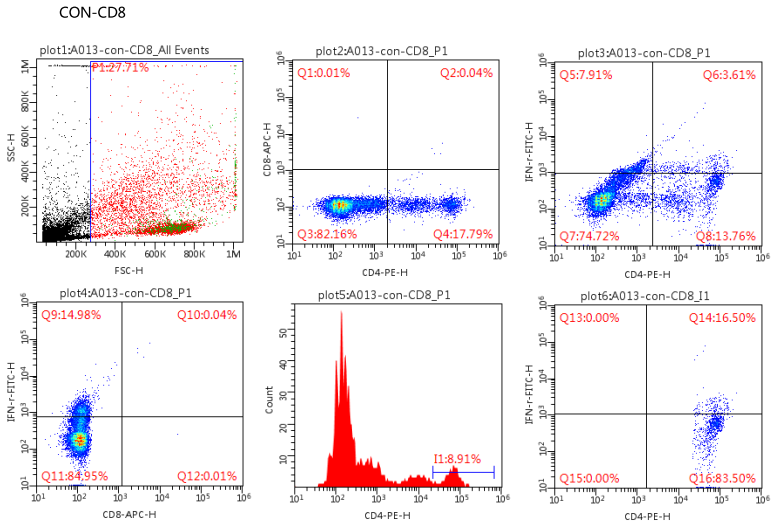


CD4-TNF-α and CD8-TNF-α expression levels of Healthy subjects (NTs)

NT-1: CD4-TNF-α and CD8-TNF-α


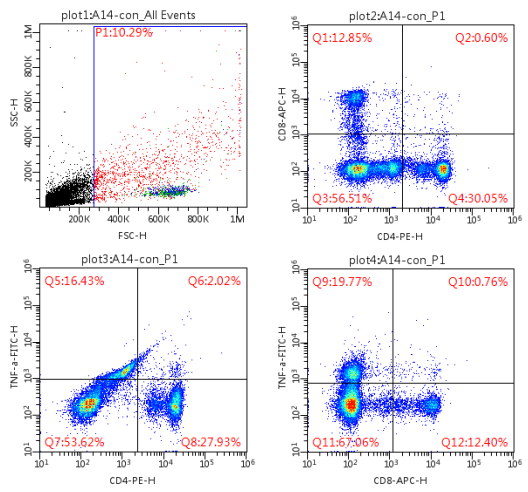


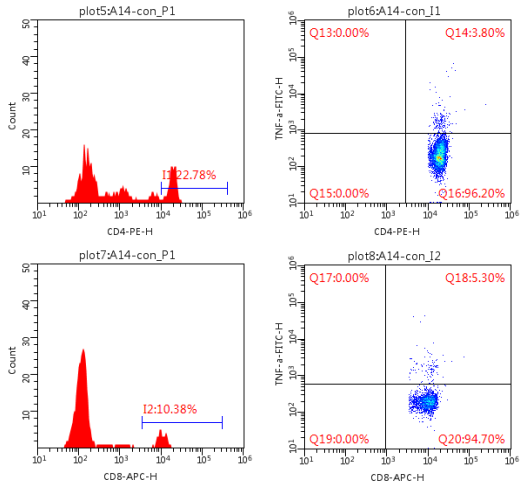


NT-2: CD4-TNF-α and CD8-TNF-α


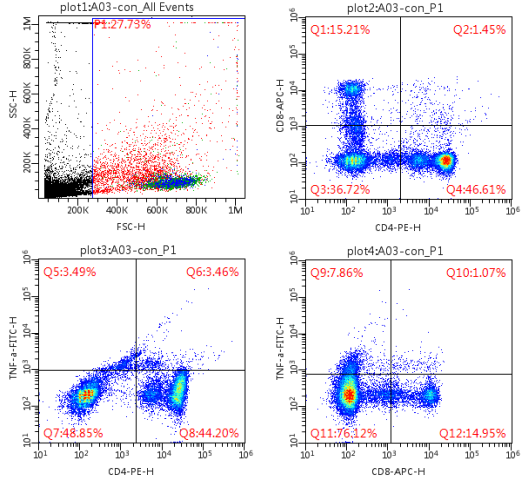


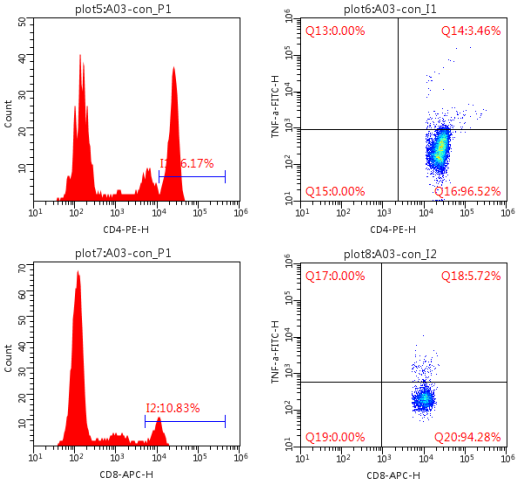


NT-3: CD4-TNF-α and CD8-TNF-α


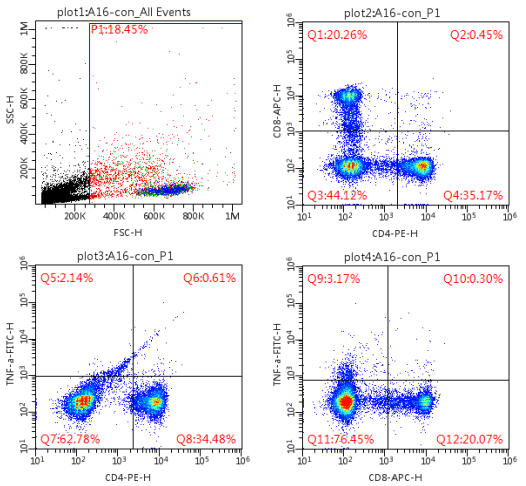


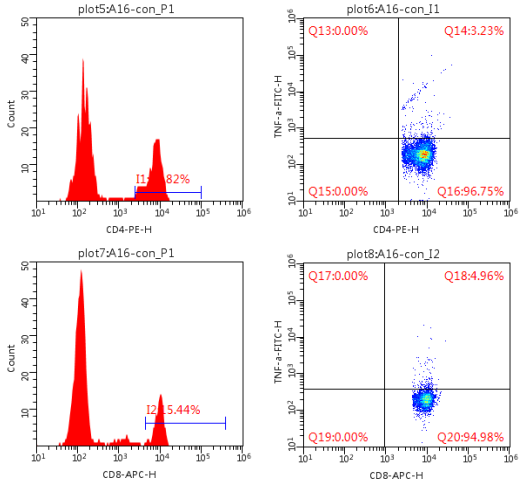


NT-4: CD4-TNF-α and CD8-TNF-α


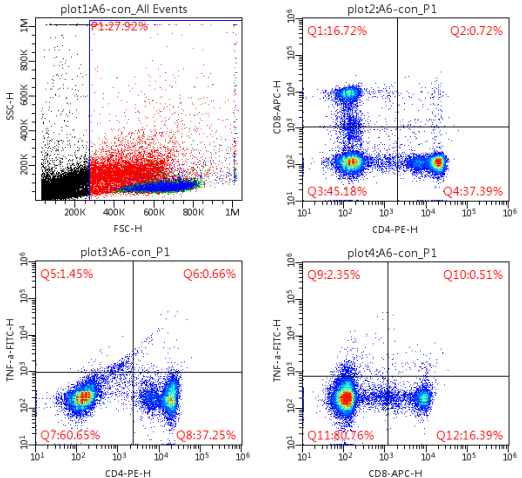


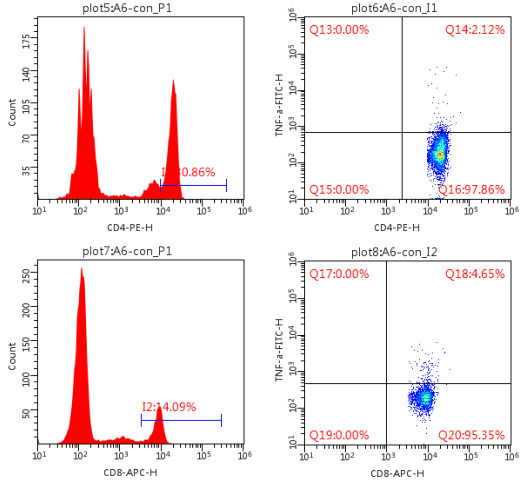


CD4-TNF-α and CD8-TNF-α expression levels of Essential hypertensive patients (EHs) EH-1: CD4-TNF-α and CD8-TNF-α


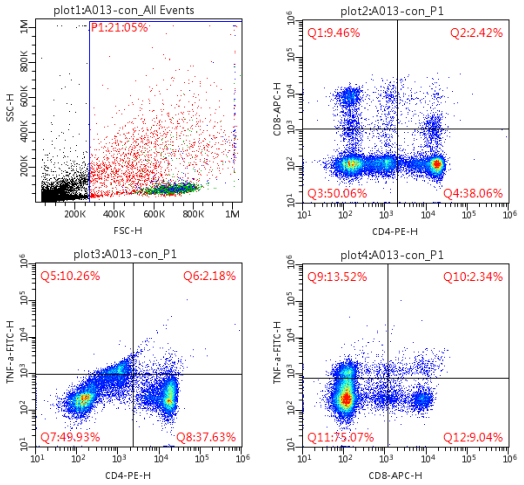


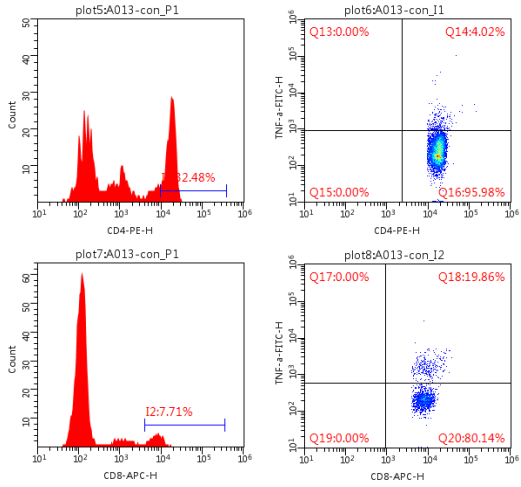


EH-2: CD4-TNF-α and CD8-TNF-α


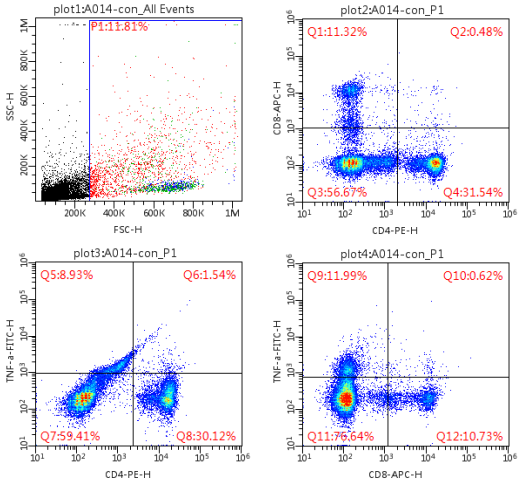


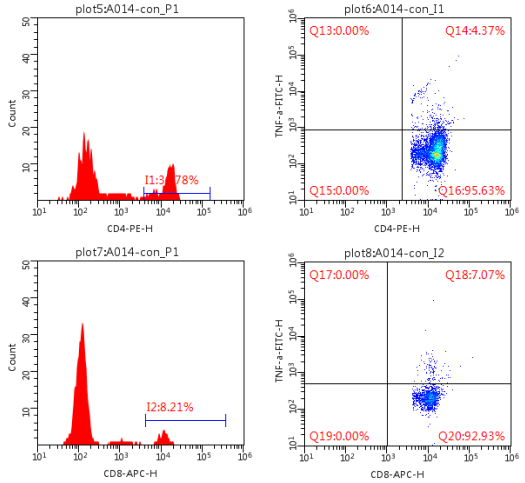


EH-3: CD4-TNF-α and CD8-TNF-α


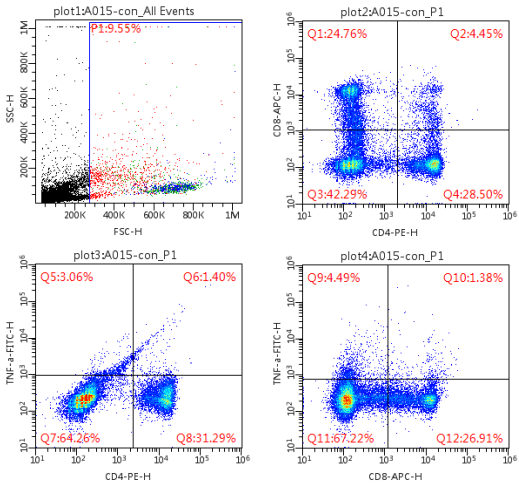


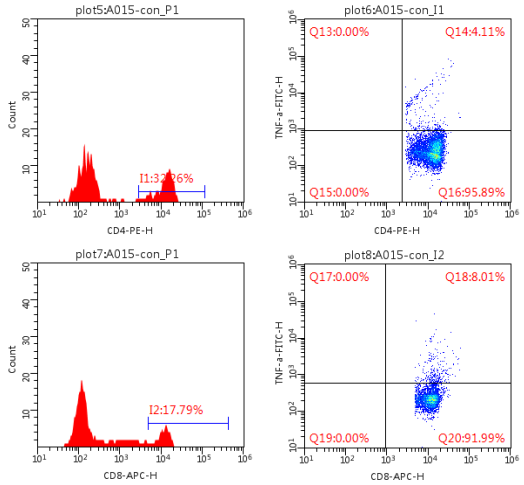

Supplement: S1 Fig — (DOCX) [file pone.0184773.s001.docx]
