# Supplementary material for: Up-regulation of gap junction in peripheral blood T lymphocytes contributes to the inflammatory response in essential hypertension
Source: PLoS One. 2017 Sep 14;12(9):e0184773. doi: 10.1371/journal.pone.0184773 (PMC5599050; doi:10.1371/journal.pone.0184773)

**S2 Fig. Flow cytometry analysis of Cxs in different T-lymphocyte subsets of healthy subjects (NTs) and essential hypertensive patients (EHs).**

CD4-Cx40 and CD8-Cx40 expressions of Healthy subjects (NTs)

NT-1: CD4-Cx40 and CD8-Cx40


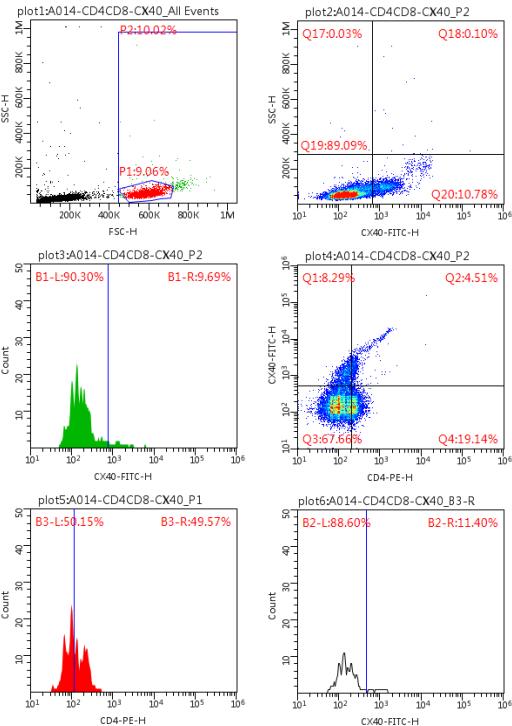


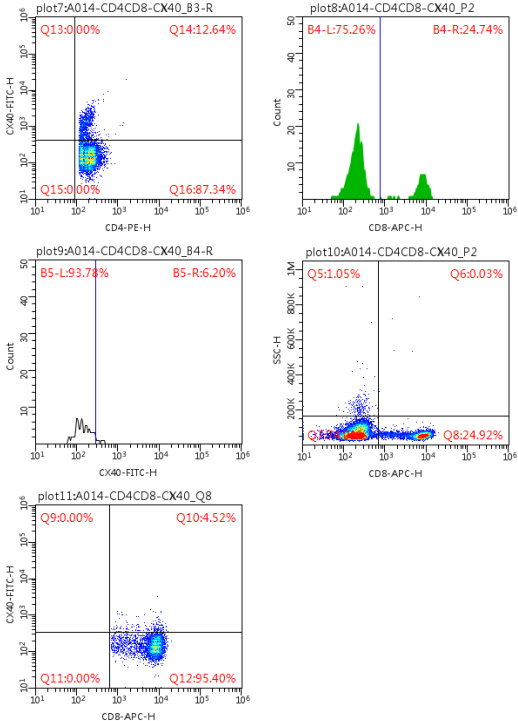


NT-2: CD4-Cx40 and CD8-Cx40


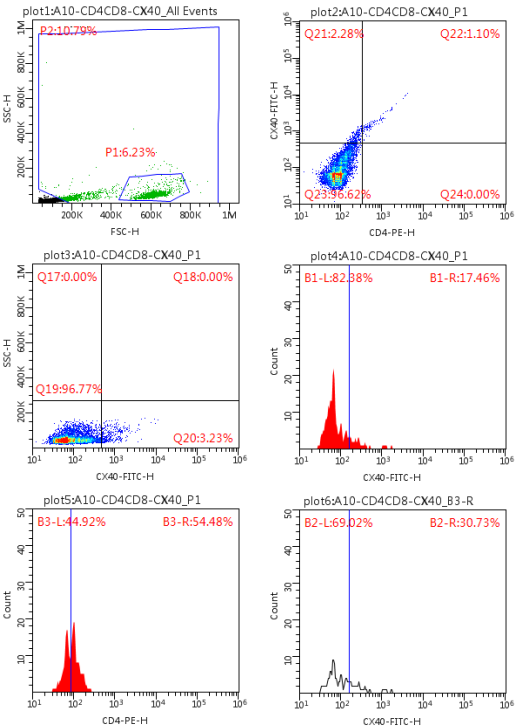


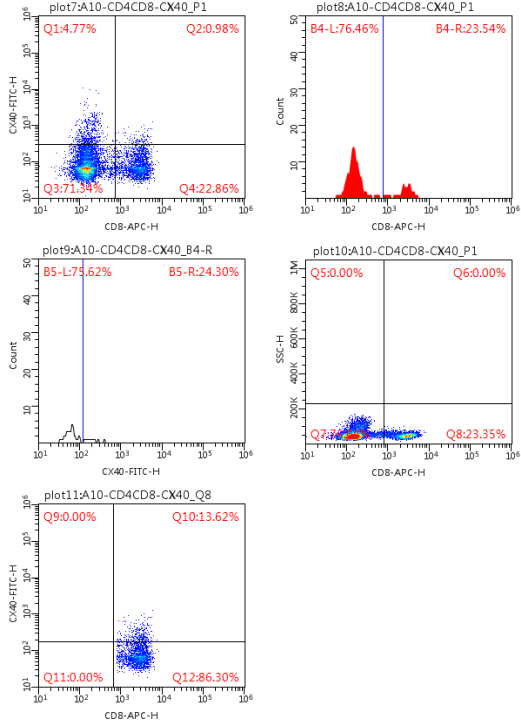


NT-3: CD4-Cx40 and CD8-Cx40


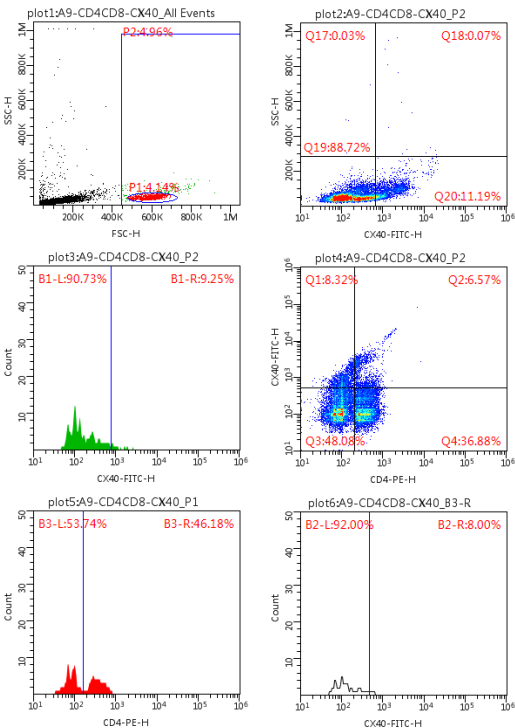


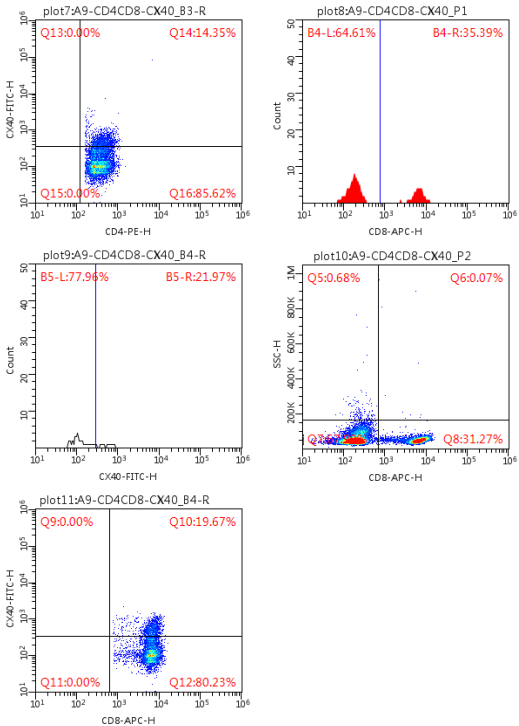


NT-4: CD4-Cx40 and CD8-Cx40


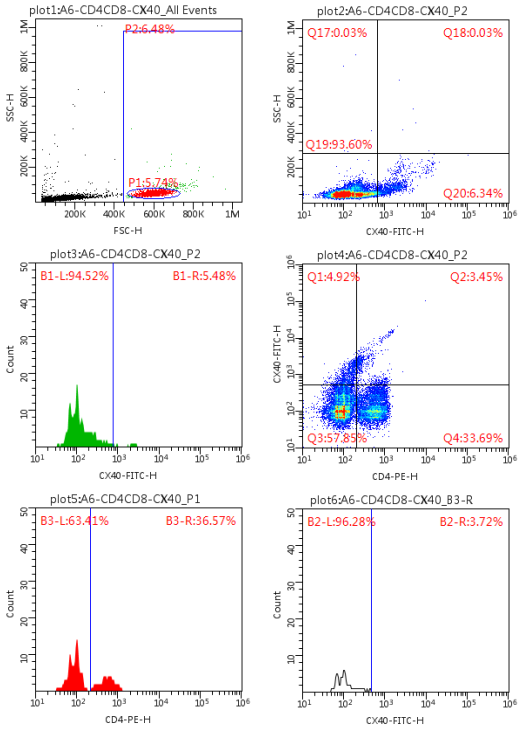


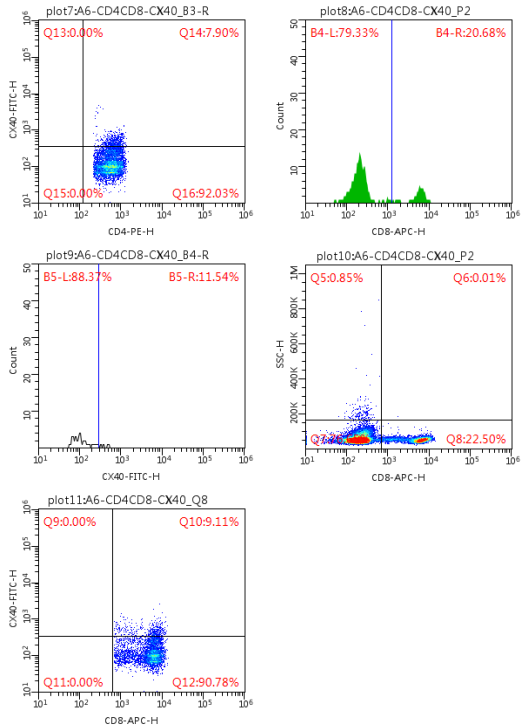


NT-5: CD4-Cx40 and CD8-Cx40


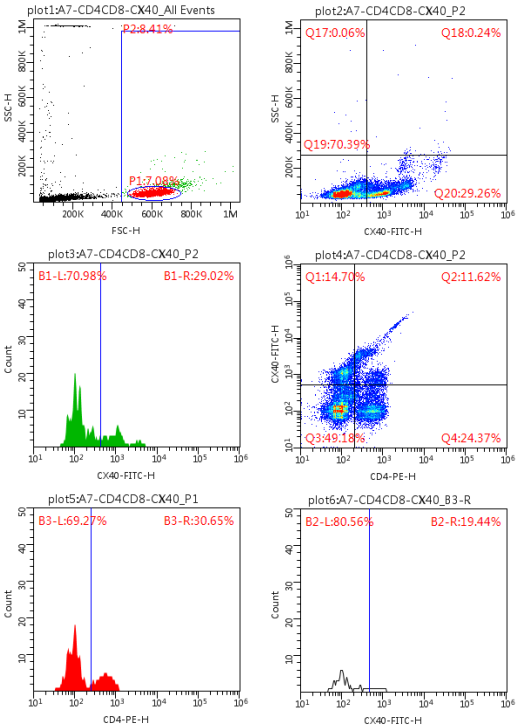


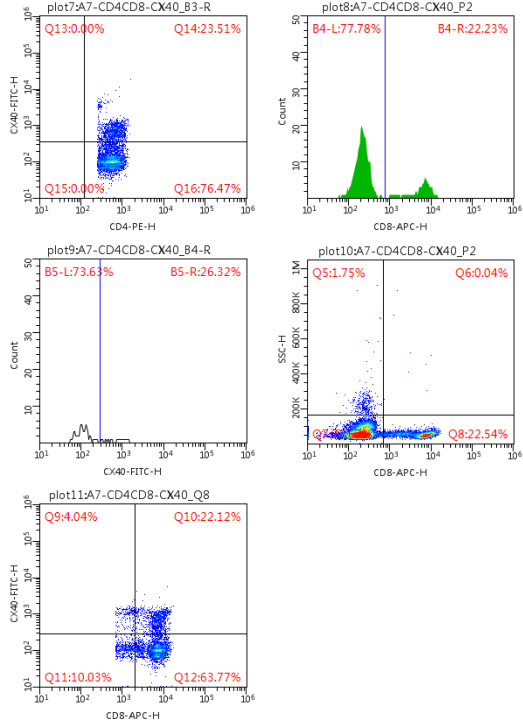


CD4-Cx40 and CD8-Cx40 expressions of Essential hypertensive patients (EHs)

EH-1: CD4-Cx40 and CD8-Cx40


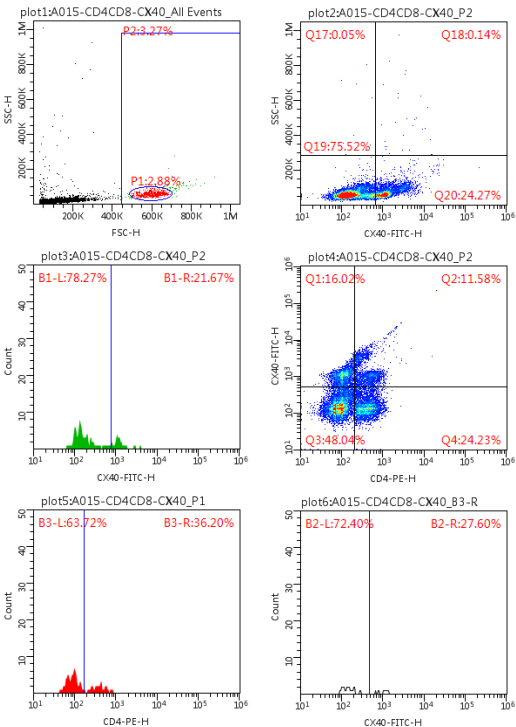


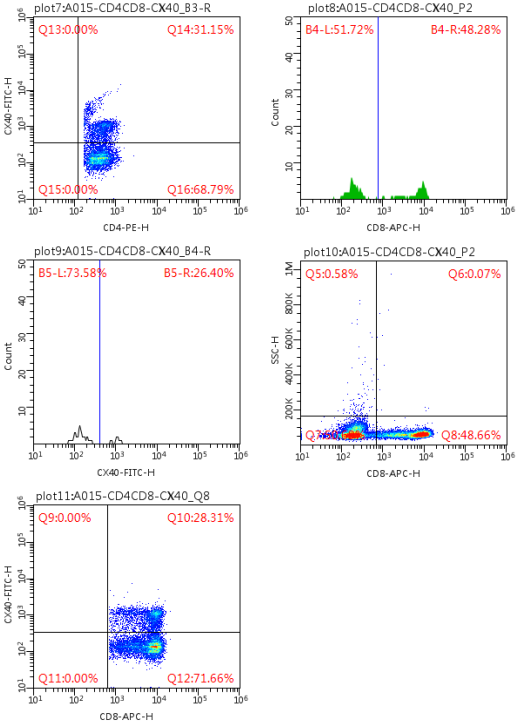


EH-2: CD4-Cx40 and CD8-Cx40


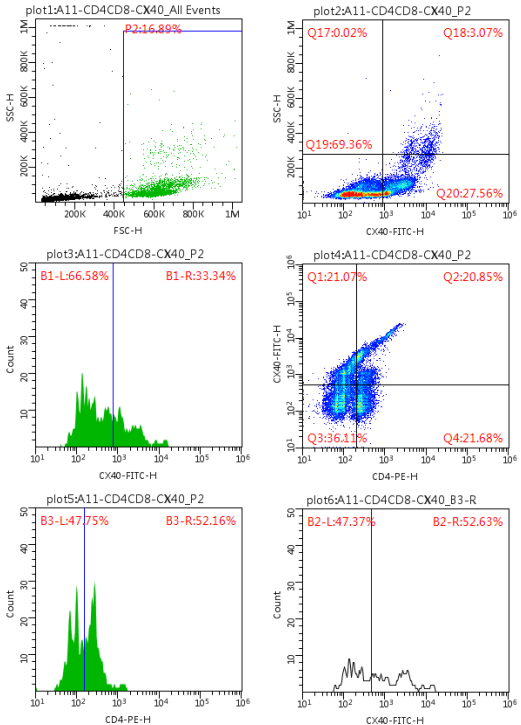


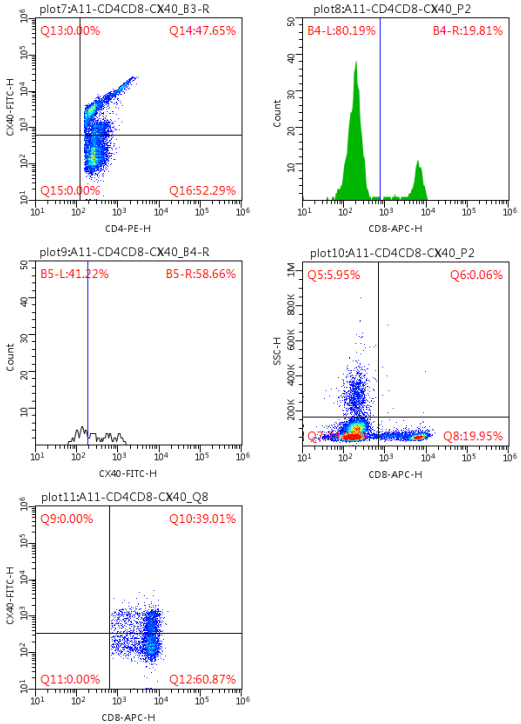


EH-3: CD4-Cx40 and CD8-Cx40


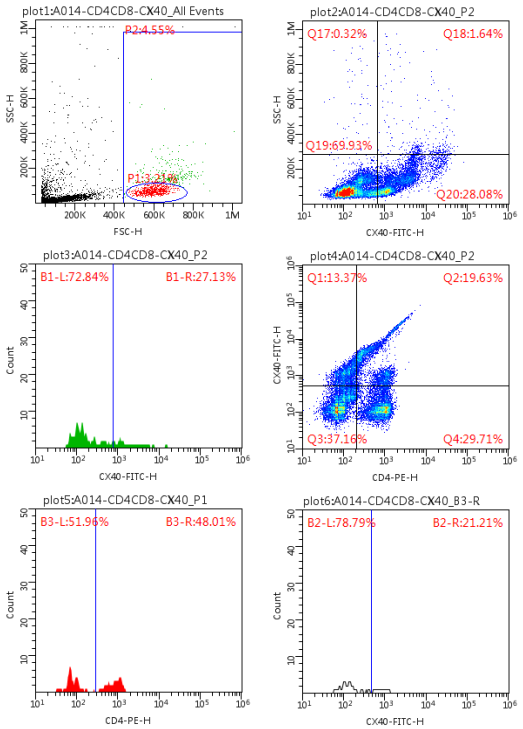


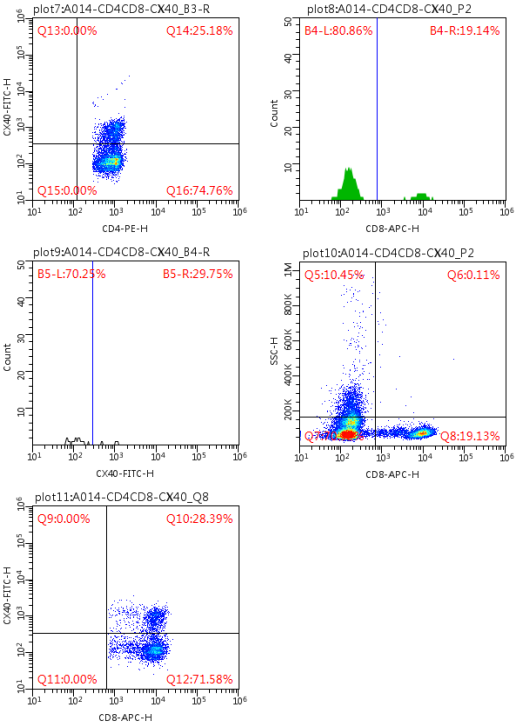


CD4-Cx43 and CD8-Cx43 expressions of Healthy subjects (NTs)

NT-1: CD4-Cx43 and CD8-Cx43


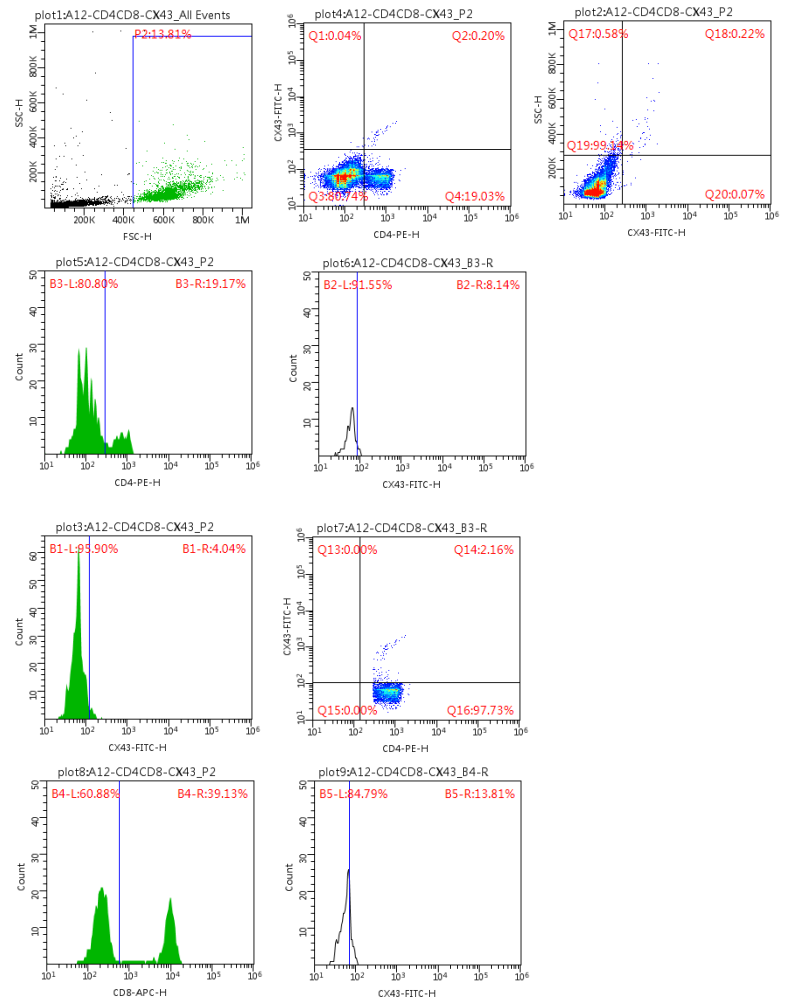


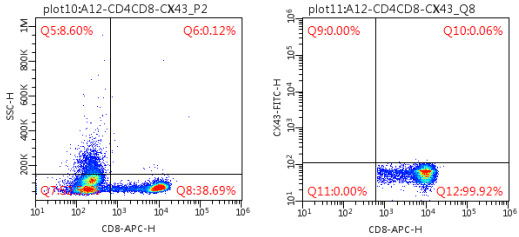


NT-2: CD4-Cx43 and CD8-Cx43


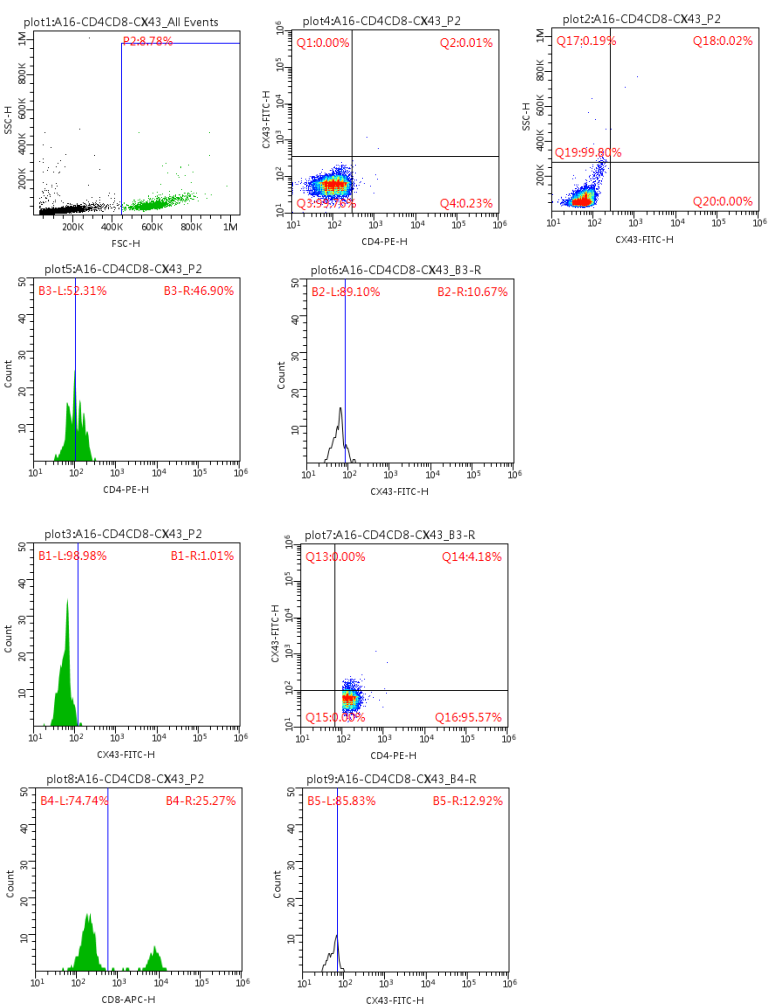


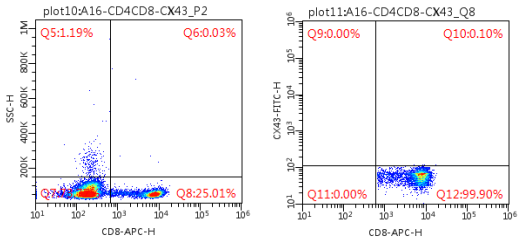


NT-3: CD4-Cx43 and CD8-Cx43


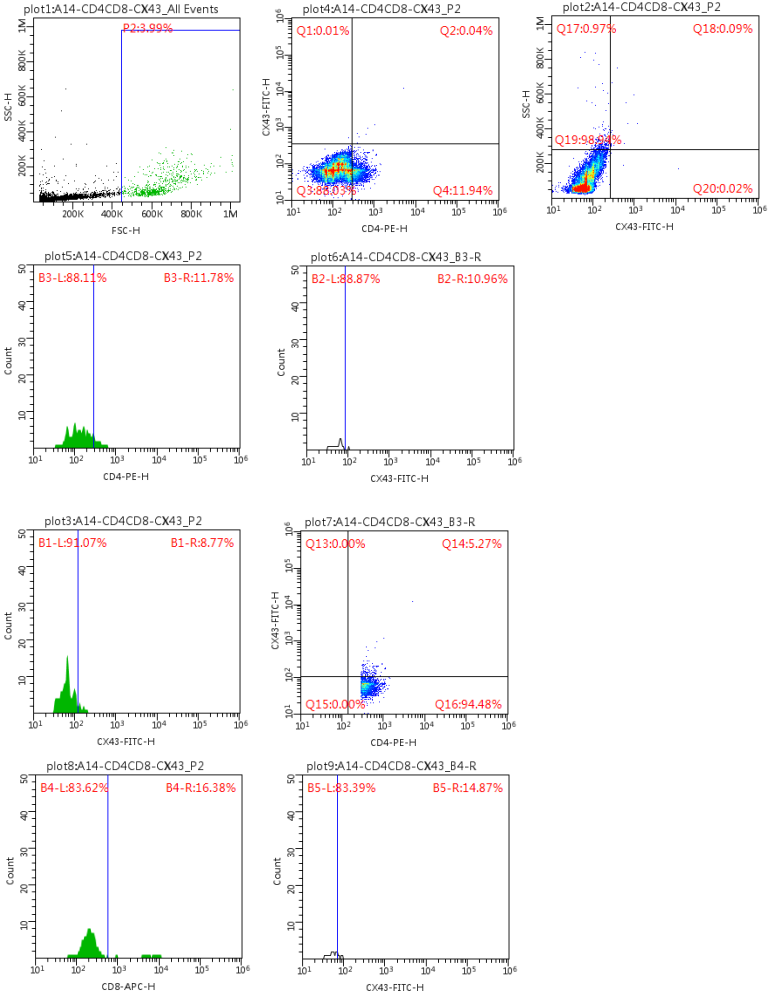


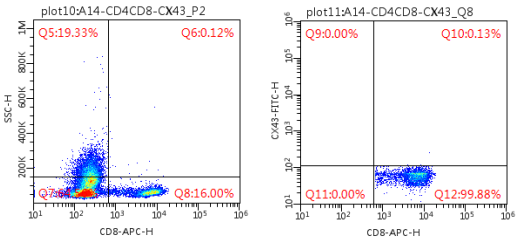


NT-4: CD4-Cx43 and CD8-Cx43


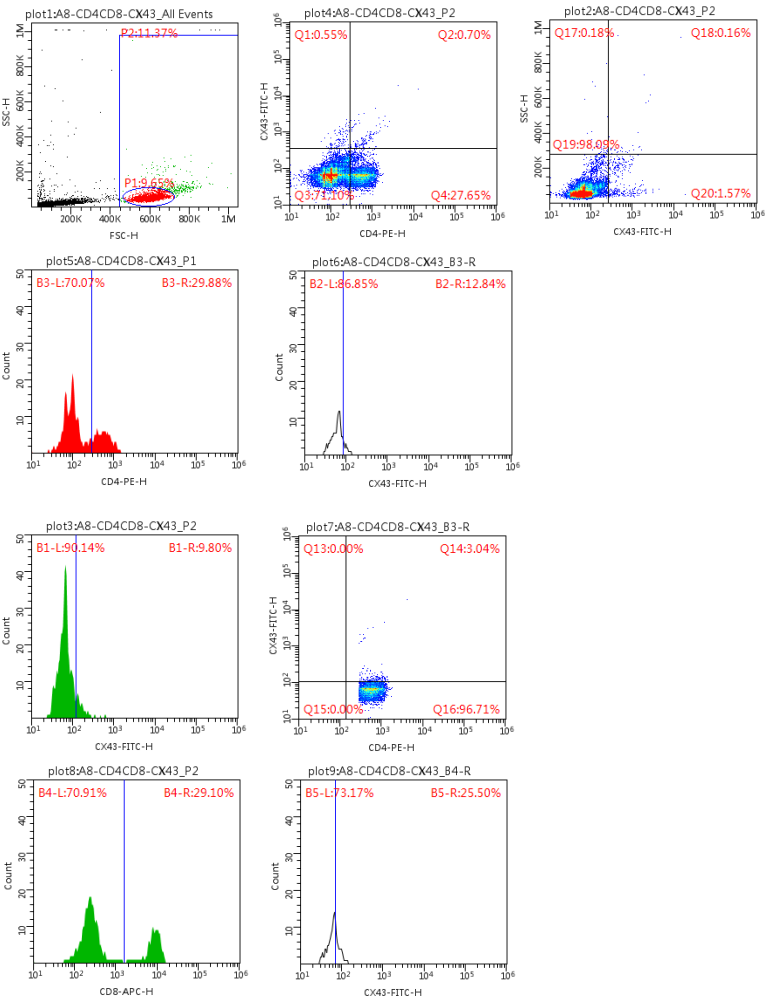


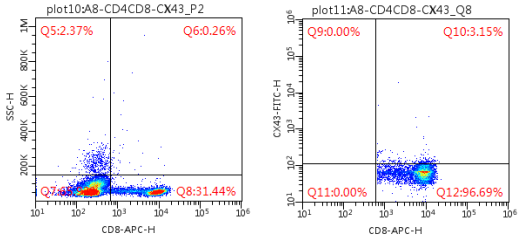


NT-5: CD4-Cx43 and CD8-Cx43


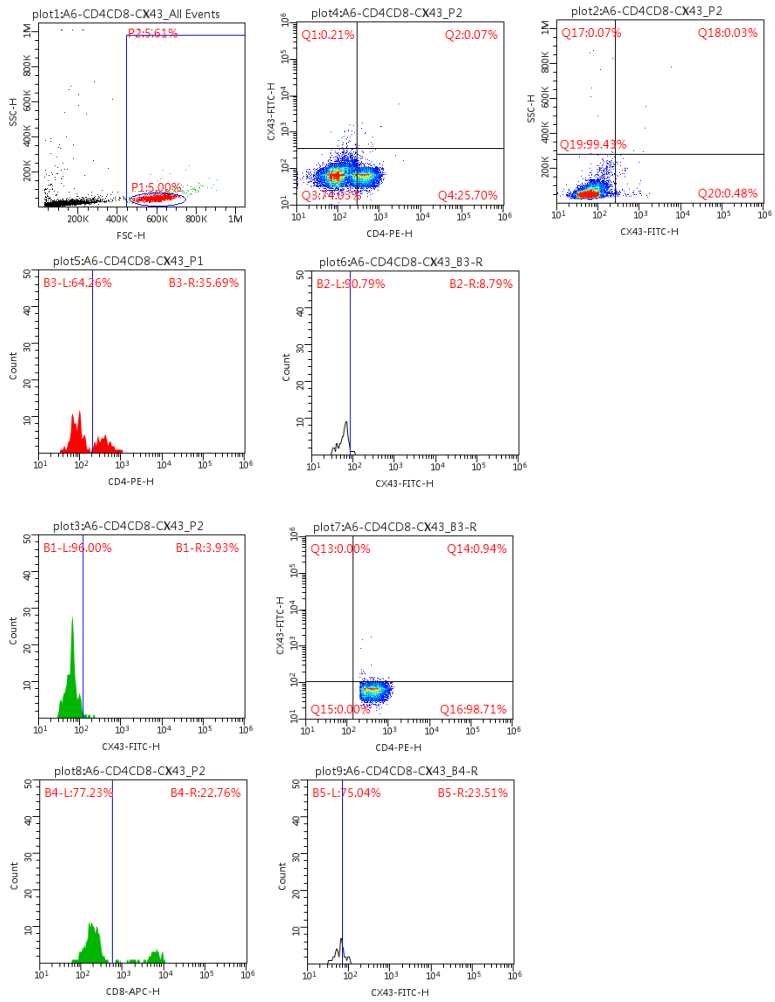


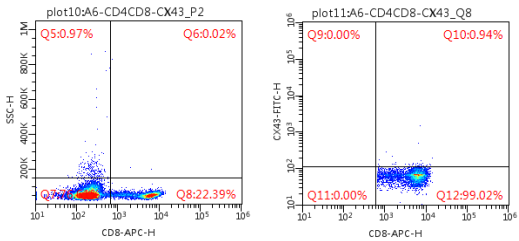


CD4-Cx43 and CD8-Cx43 expressions of Essential hypertensive patients (EHs)

EH-1: CD4-Cx43 and CD8-Cx43


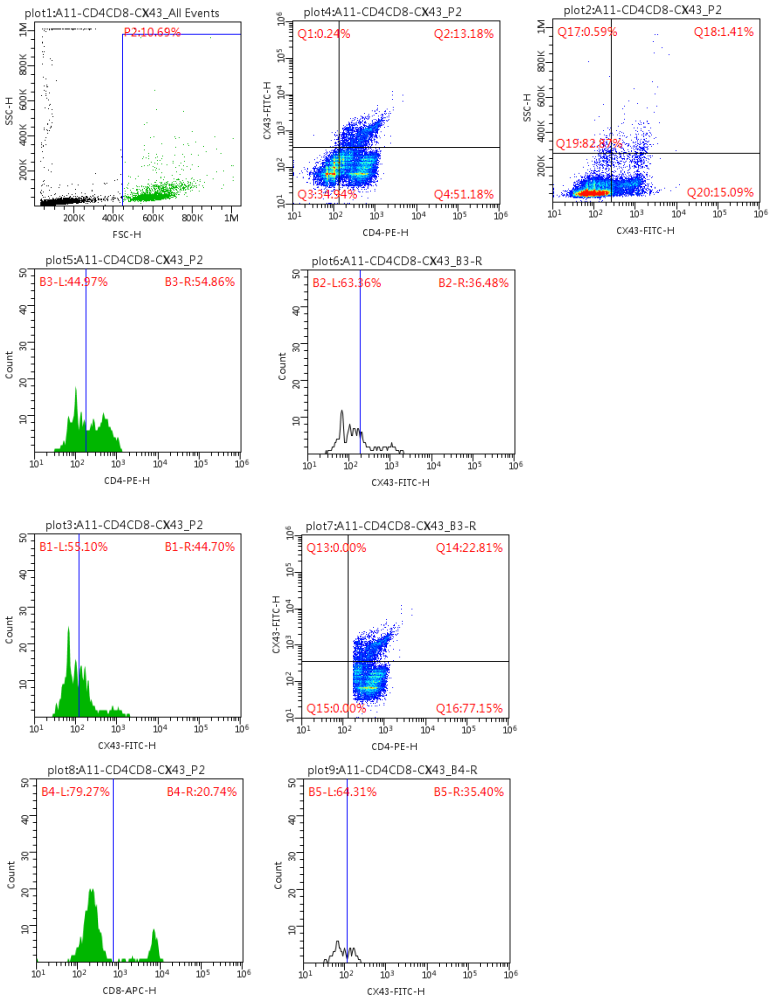


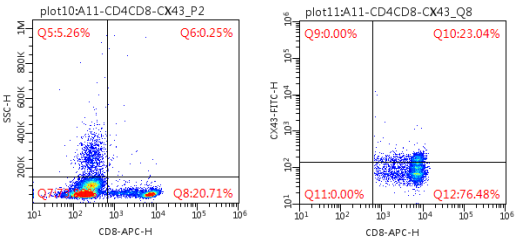

Supplement: S2 Fig — (DOCX) [file pone.0184773.s002.docx]
