# Supplementary material for: Up-regulation of gap junction in peripheral blood T lymphocytes contributes to the inflammatory response in essential hypertension
Source: PLoS One. 2017 Sep 14;12(9):e0184773. doi: 10.1371/journal.pone.0184773 (PMC5599050; doi:10.1371/journal.pone.0184773)

**Functional experiment of gap junction from peripheral blood lymphocytes of Healthy subjects (NTs) and Essential hypertensive patients (EHs)**

1. Control experiment, including no label, DiIC_18_ positive lymphocytes, Calcein-AM positive lymphocytes and DiIC_18_-Calcein double-labelled lymphocytes


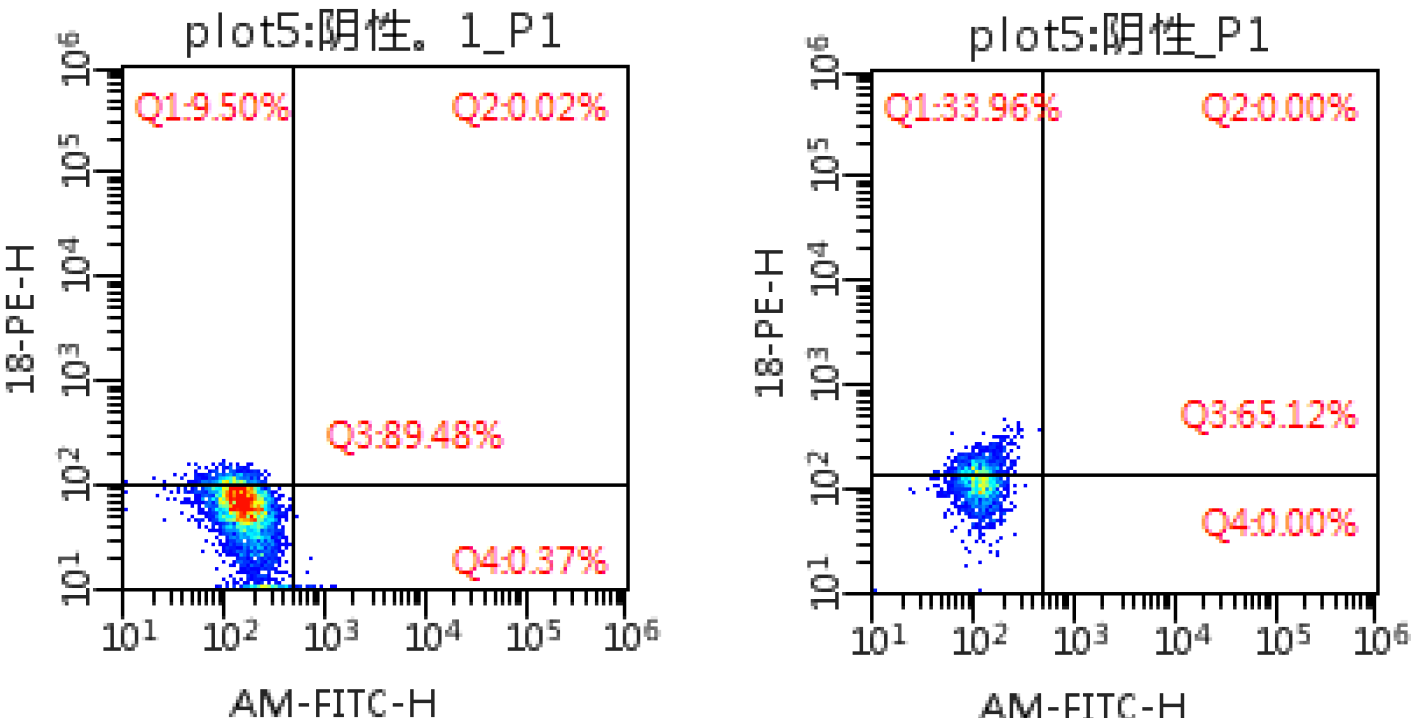


no label


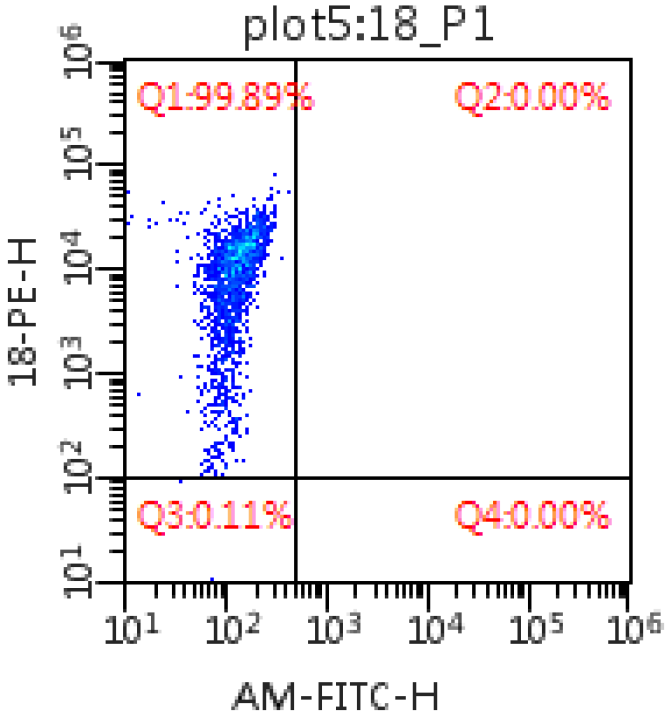


DiIC_18_ positive lymphocytes


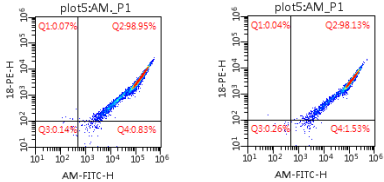


Calcein-AM positive lymphocytes


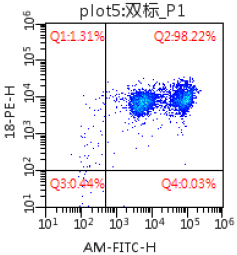


DiIC_18_-Calcein double-labelled lymphocytes

1. Gap junctional intercellular communication in peripheral blood lymphocytes from Healthy subjects (NTs)

Control IL-2 Gap27+IL-2


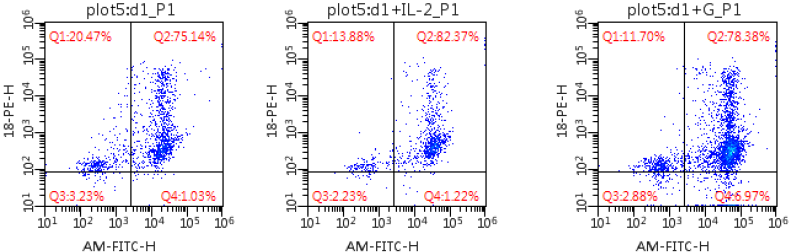


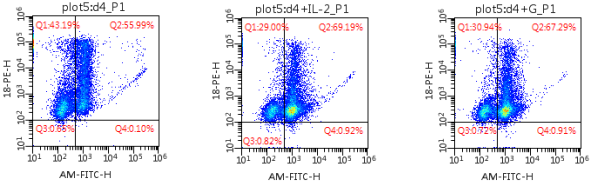


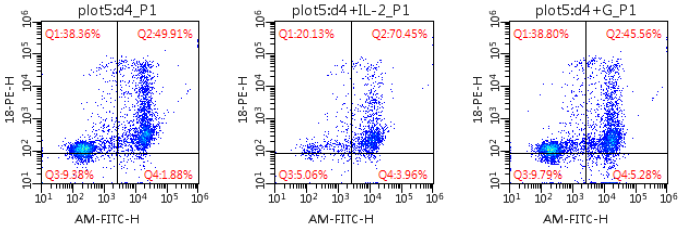


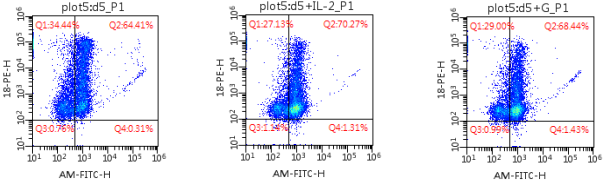


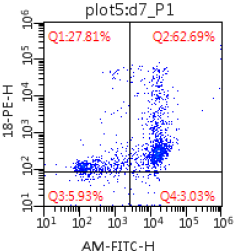

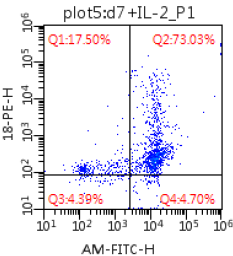

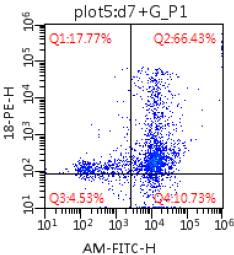


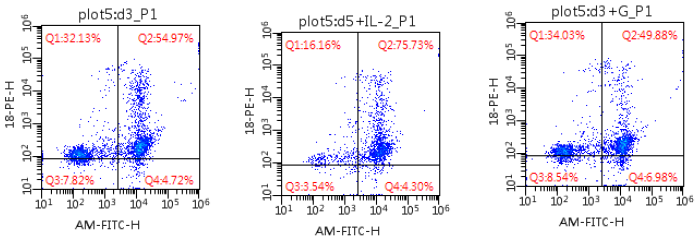


1. Gap junctional intercellular communication in peripheral blood lymphocytes from Essential hypertensive patients (EHs)

Control IL-2 Gap27+IL-2


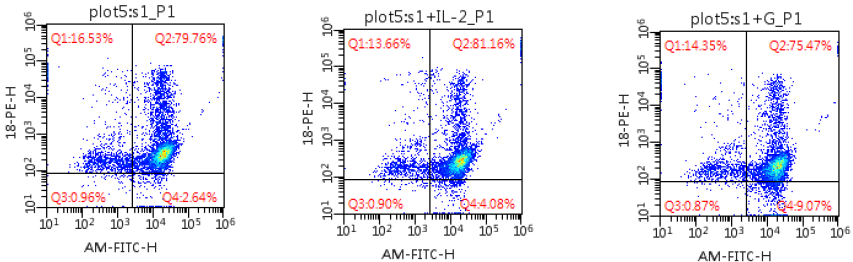


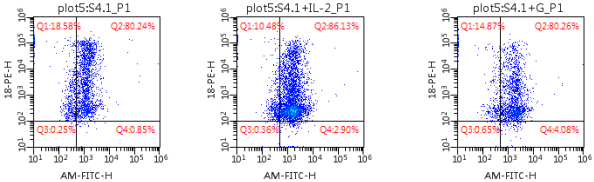


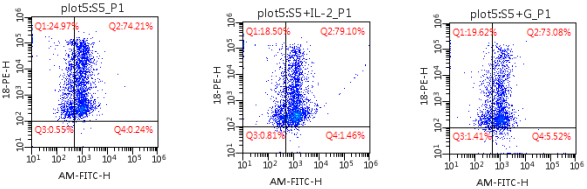


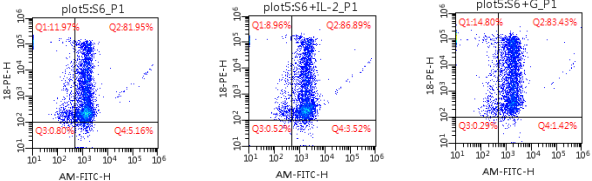

Supplement: S3 Fig — (DOCX) [file pone.0184773.s003.docx]
